# Inflammation-sensing catalase-mimicking nanozymes alleviate acute kidney injury via reversing local oxidative stress

**DOI:** 10.1186/s12951-022-01410-z

**Published:** 2022-04-27

**Authors:** Hong Sang Choi, Ansuja Pulickal Mathew, Saji Uthaman, Arathy Vasukutty, In Jin Kim, Sang Heon Suh, Chang Seong Kim, Seong Kwon Ma, Sontyana Adonijah Graham, Soo Wan Kim, In-Kyu Park, Eun Hui Bae

**Affiliations:** 1 Departments of Internal Medicine, Chonnam National University Medical School, 160, Baekseo‑ro, Dong‑gu, Gwangju, 61469 Republic of Korea; 2 Departments of Internal Medicine, Chonnam National University Hospital, Gwangju, Republic of Korea; 3 Department of Biomedical Sciences, BK21 PLUS Center for Creative Biomedical Scientists, Chonnam National University Medical School, 160, Baekseo‑ro, Dong‑gu, Gwangju, 61469 Republic of Korea; 4 BioMedical Sciences Graduate Program (BMSGP), Chonnam National University, Hwasun-gun, Jeollanam-do Republic of Korea

**Keywords:** Mn_3_O_4_ nanoparticles, Nanozymes, Inflammation, Ischemia–reperfusion, Kidney

## Abstract

**Background:**

The reactive oxygen species (ROS) and inflammation, a critical contributor to tissue damage, is well-known to be associated with various disease. The kidney is susceptible to hypoxia and vulnerable to ROS. Thus, the vicious cycle between oxidative stress and renal hypoxia critically contributes to the progression of chronic kidney disease and finally, end-stage renal disease. Thus, delivering therapeutic agents to the ROS-rich inflammation site and releasing the therapeutic agents is a feasible solution.

**Results:**

We developed a longer-circulating, inflammation-sensing, ROS-scavenging versatile nanoplatform by stably loading catalase-mimicking 1-dodecanethiol stabilized Mn_3_O_4_ (dMn_3_O_4_) nanoparticles inside ROS-sensitive nanomicelles (PTC), resulting in an ROS-sensitive nanozyme (PTC-M). Hydrophobic dMn_3_O_4_ nanoparticles were loaded inside PTC micelles to prevent premature release during circulation and act as a therapeutic agent by ROS-responsive release of loaded dMn_3_O_4_ once it reached the inflammation site.

**Conclusions:**

The findings of our study demonstrated the successful attenuation of inflammation and apoptosis in the IRI mice kidneys, suggesting that PTC-M nanozyme could possess promising potential in AKI therapy. This study paves the way for high-performance ROS depletion in treating various inflammation-related diseases.

**Graphical Abstract:**

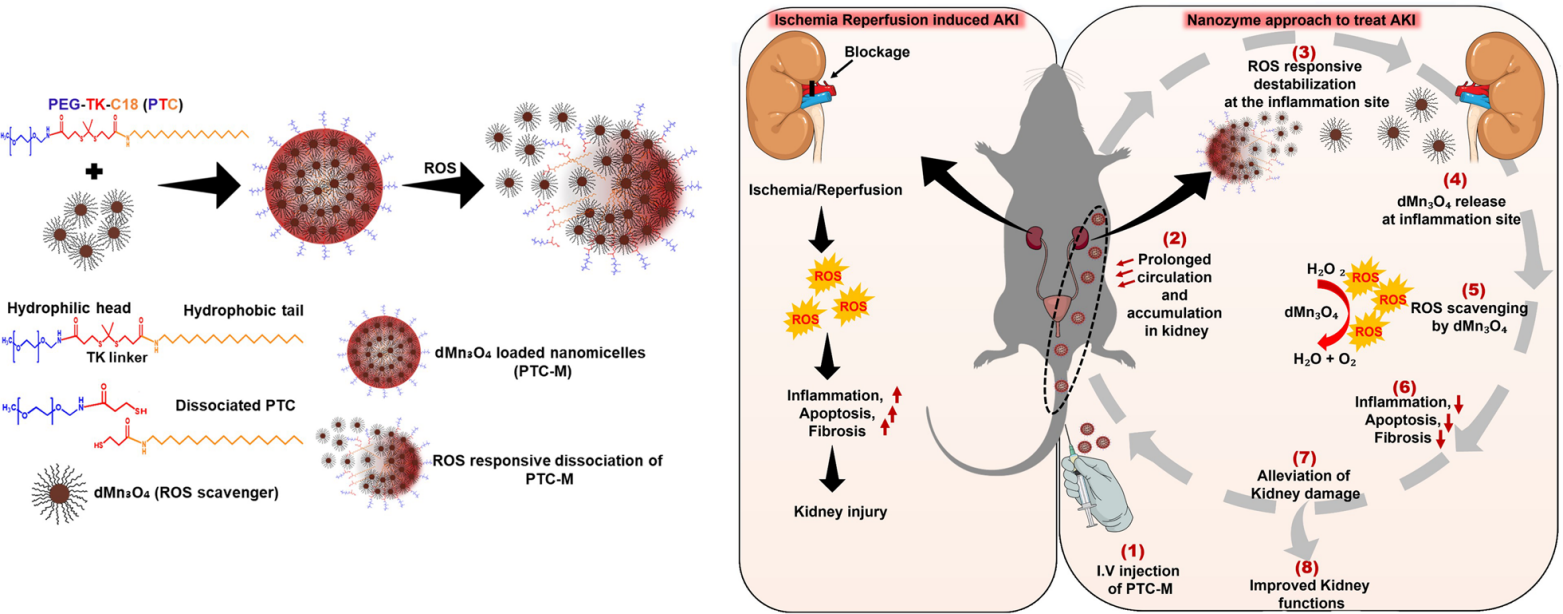

**Supplementary Information:**

The online version contains supplementary material available at 10.1186/s12951-022-01410-z.

## Background

Acute kidney injury (AKI) is the term that covers a broad spectrum of renal function deterioration ranging from small changes in serum creatinine level to conditions requiring renal replacement therapy [1]. The KDIGO (Kidney disease: improving global outcomes) guidelines define AKI as an increase in serum creatinine of either > 0.3 mg/dL within 48 h, > 1.5 times from baseline within seven days, or a reduction in urine volume of < 0.5 ml/kg/hour for six hours [1]. AKI is one of the rising global health problems that causes with high morbidity and mortality and economic burden [2]. The incidence of AKI defined by the KDIGO guidelines was 21.6% in adults in in-hospital settings [3]. AKI may lead to several complications, including metabolic acidosis, hyperkalemia, uremia, volume overload, and damage to other organ systems, including death. Despite advances in medicine, mortality from AKI is still high. According to a meta-analysis of studies in in-hospital settings, AKI-associated mortality was reported to be 23.9% in adults [3]. The mortality rate of dialysis-requiring AKI patients exceeds 50% [4]. AKI increases the long-term risk of subsequent AKI, progressive chronic kidney disease (CKD), and end-stage renal disease (ESRD) [5]. Eventually, AKI increases socioeconomic and healthcare burdens. Nevertheless, there is no clear treatment for AKI to date. When AKI occurs, conservative treatments such as removing the causative factors, controlling water and electrolyte imbalances, and performing renal replacement therapy, if necessary, and waiting for renal function to recover are applied.

Ischemia–reperfusion injury (IRI) is organ injury caused by the transient reduction or cessation of blood flow, followed by reperfusion, and can be encountered in a variety of clinical situations, such as shock, myocardial infarction, ischemic stroke, major surgery, and organ transplantation. Renal IRI is one of the leading causes of AKI, which results in a rapid decline in renal function. The kidney is an organ that is particularly susceptible to ischemic insults and injured tubular epithelial cells consequently cause impaired waste product excretion, and water, electrolyte, and acid–base balance control [6]. The mechanism of renal tubular cell injury in IRI is known to involve inflammation caused by reactive oxygen species (ROS) generated during reperfusion [6–8]. Therefore, there have been attempts to apply ROS scavenging to the treatment of IRI. However, its real clinical use has been limited due to concerns for the systemic side effects, which include hypersensitivity reactions [9]. The administration of antioxidants such as edaravone has already been used to scavenge ROS in IRI. However, it was accompanied by several side effects, and hence, demands for new and effective antioxidants such as nanozymes (nanomaterials exhibiting natural enzyme-like activity) have been made [9–13]. Yao et al. found that Mn_3_O_4_ nanoparticles possessed multiple enzyme-mimicking activities, i.e., superoxide dismutase and catalase-mimicking activities as well as hydroxyl radical scavenging activity [11]. It was shown that Mn_3_O_4_ possessed a stronger ROS-scavenging ability than other nanozymes such as V_2_O_5_, CeO_2_, Co_3_O_4_, MnFe_2_O_4_, and Fe_3_O_4_ and was better than that of other manganese oxide types (MnO_x_ (MnO_2_, MnO, and Mn_2_O_3_).

**Fig. 1:**
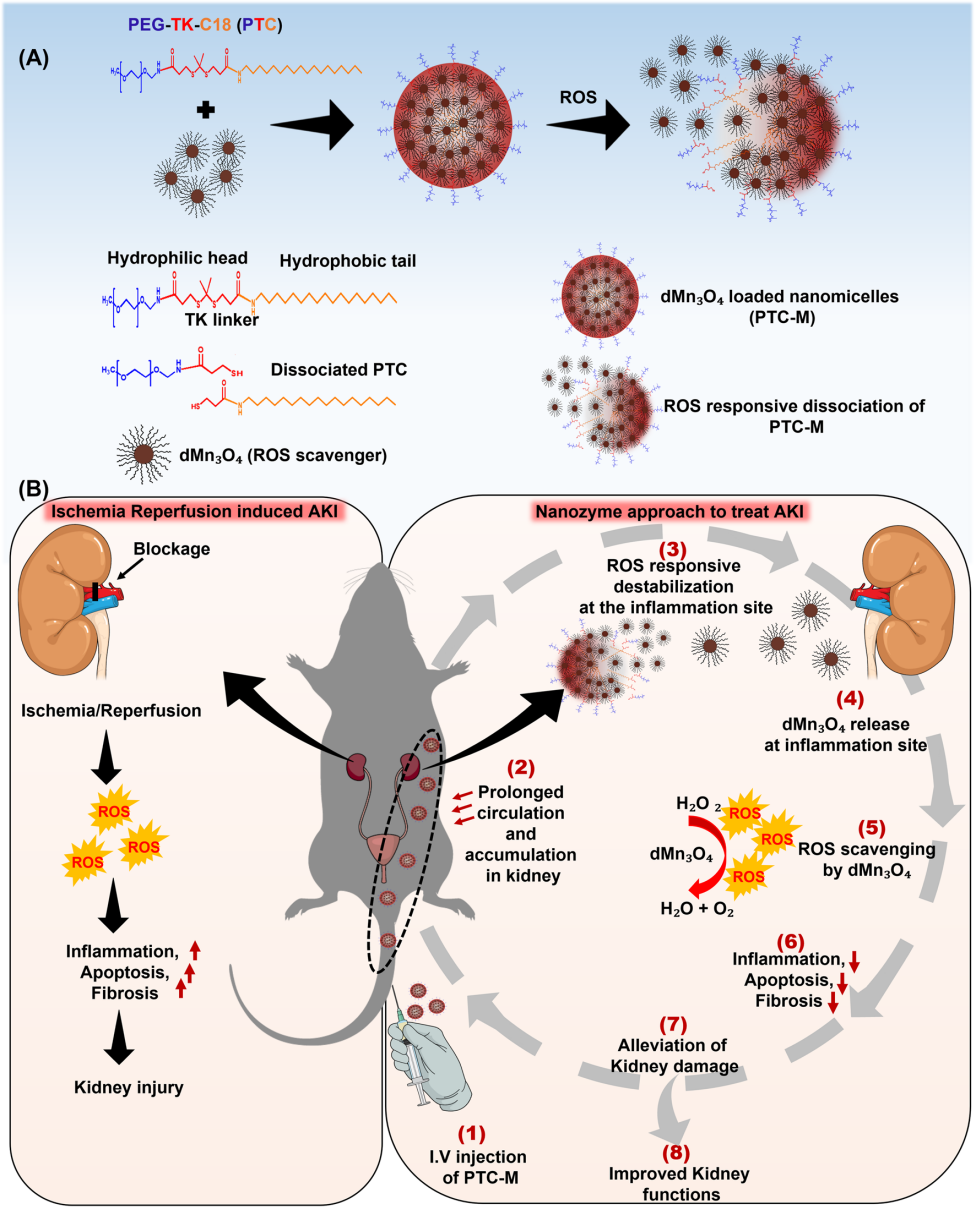
Schematic illustration showing preparation and mode of action of PTC-M **A** Synthesis of PTC-M, **B** Mechanism of action of PTC-M (1) intravenous injection of PTC-M (2) the injected PTC-M circulates in blood and accumulates at the inflammation site (3) the destabilization of PTC-M due to the intrinsic ROS present at the inflammation site (4) release of the loaded dMn_3_O_4_ at the inflammation site (5) the released dMn_3_O_4_ catalyzes the ROS scavenging leading to (6) reduction in inflammation, apoptosis and fibrosis (7) alleviation of kidney damage and (8) improved kidney functions

In this work, we developed an inflammation-sensing, ROS-scavenging versatile nanoplatform by stably loading catalase-mimicking dMn_3_O_4_ nanoparticles inside ROS-sensitive nanomicelles (PTC), resulting in an ROS-sensitive nanozyme (PTC-M) (Fig. 1). We tried to determine if the administration of the ROS-sensitive nanozyme to the renal IRI mouse model could revert the damage caused by ROS. To the best of our knowledge, 1-dodecanethiol stabilized hydrophobic Mn_3_O_4_ nanoparticles (dMn_3_O_4_ NPs), as well as the application of dMn_3_O_4_ NPs in kidney disease, in vivo has not been reported elsewhere. We utilized the ROS-scavenging activity of the novel dMn_3_O_4_ NPs to treat kidney damage. Unlike the traditional drug administration methods, the ROS-sensitive nanomicelles could deliver the dMn_3_O_4_ NPs to the target organ more precisely and with fewer systemic side effects [14].

## Results

### Synthesis and characterization of dMn_3_O_4_ nanoparticles and polymer conjugates

**Fig. 2:**
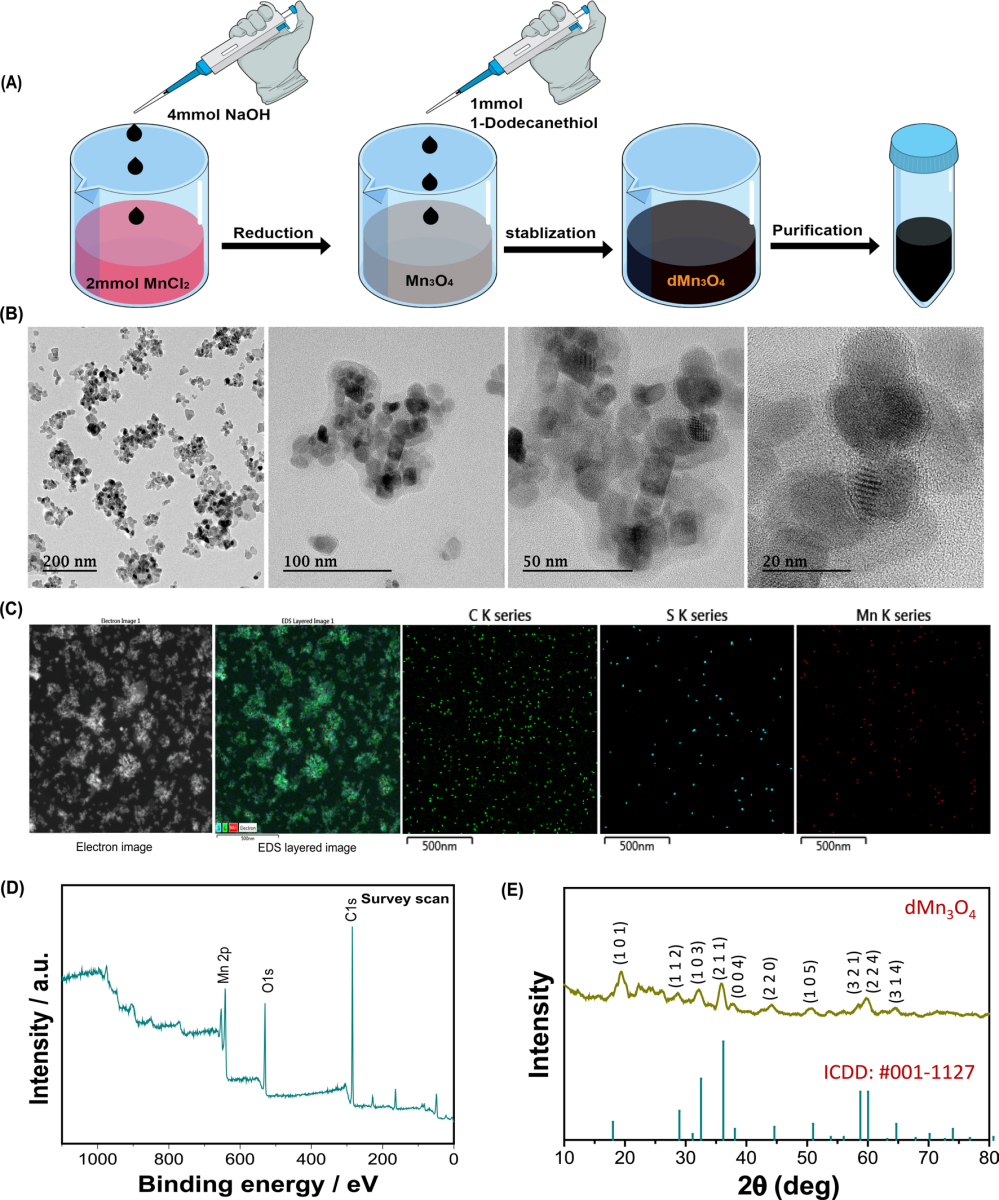
Synthesis and characterization of dMn_3_O_4_ **A** schematic illustration of dMn_3_O_4_ synthesis, **B** FE-TEM images, **C** EDS analysis, **D** XPS spectrum and **E** XRD patterns of dMn_3_O_4_ NPs

The dMn_3_O_4_ NPs were synthesized using the coprecipitation method and stabilized using thiol groups of 1-dodecanethiol (Fig. 2A). Field-emission transmission electron microscopy (FE-TEM) analysis demonstrated that dMn_3_O_4_ NPs had an average size less than 20 nm (18.0 ± 0.6 nm) (Fig. 2B). Additional file 1: Figure S1A and S1B show the images and schematic representation of the prepared dMn_3_O_4._ The Energy dispersive X-ray spectroscopy (EDS) data showed the presence of Mn from Mn_3_O_4_ and sulfur and carbon from SH-C12 (Fig. 2C and Additional file 1: Figure S1C). The X-ray photon spectroscopy (XPS) analysis results depicted the XPS spectrum of Mn 2*p*, which consisted of two peaks located at 641.42 eV and 653.23 eV and these peaks were attributed to Mn 2*p*_3/2_ and Mn 2*p*_1/2_, respectively (Fig. 2D and Additional file 1: Figure S2). The energy gap between these two peaks was 11.81 eV, which was in good agreement with the reported literature for Mn_3_O_4_. In the X-ray powder diffraction (XRD) pattern of the dMn_3_O_4_ NPs, as shown in Fig. 2E, all the measured diffraction peaks matched well to the standard pattern of hausmannite Mn_3_O_4_ [ICDD: #001–1127], confirming their highly crystalline nature. The ROS-responsive PTC conjugate was prepared via stepwise chemical reactions where the thioketal (TK) linker with a terminal carboxylate group was synthesized first. The carboxylic group of the TK linker was then conjugated to the amino group of Methoxy-PEG amine (PEG-AM) using the EDC/NHS reaction to form PEG-TK. Finally, the other carboxylic group of PEG-TK was conjugated to the amino group of stearamine again via the EDC/NHS reaction [15]. The ROS nonresponsive PC conjugate was prepared by conjugating stearic acid to PEG-AM. The composition of PTC and PC was analyzed by ^1^H nuclear magnetic resonance (NMR) spectroscopy (Additional file 1: Figure S3). The NMR spectra showed characteristic peaks at δ 0.83 and 1.23 ppm corresponding to the methyl and methylene protons of C18. The peak at δ 3.6 corresponded to O–CH2–CH2- of PEG. The degree of substitution (DS %) of C18 in PTC and PC was calculated as 78% and 46%, respectively.

### Preparation and characterization of PTC-M nanozyme

**Fig. 3:**
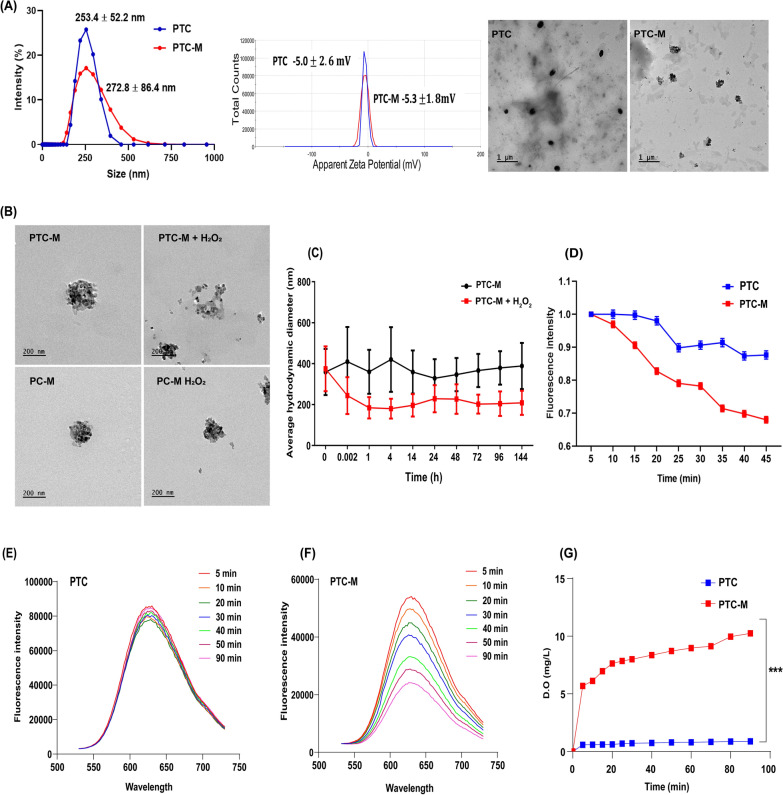
Synthesis and characterization of PTC-M **A** Hydrodynamic diameter, zeta potential distribution and FE-TEM images of PTC and PTC-M, **B** Size difference of PTC-M and PC-M treated with 1 mM H_2_O_2_, **C** Diameter change of PTC-M and H_2_O_2_ treated PTC-M up to 6 days, **D** H_2_O_2_ scavenging assay using terephthalic acid, **E** Fluorescence spectra of Ru(ddp) in PTC and **F** PTC-M treated with H_2_O_2_ (1 mM) at different incubation times, **G** Dissolved oxygen generation by PTC and PTC-M treated with H_2_O_2_ (1 mM). Data is shown as mean ± SEM (n = 3 replicates). Statistics were performed by a one-way ANOVA (*P < 0.05, **P < 0.01, ***P < 0.001, and ****P < 0.0001)

The ROS-sensitive nanomicelles (PTC) were loaded with dMn_3_O_4_, resulting in PTC-M. The dMn_3_O_4_ feed ratio (20%) of PTC was utilized for the preparation of PTC-M. The hydrodynamic diameter, zeta potential, and FE-TEM images of the prepared PTC and PTC-M are shown in Fig. 3A and Additional file 1*:* Figure S4A. The FE-TEM images of PTC demonstrated spherical nanomicelles with uniform particle sizes. The hydrodynamic diameter of PTC-M was slightly increased after the loading of dMn_3_O_4_ because of the packing of dMn_3_O_4_ in the hydrophobic space while the zeta potential stayed negative in both nanomicelles due to PEG shielding. The FE-TEM images of PTC and PTC-M showed uniformly distributed particles. The loading content and encapsulation efficiency were calculated using mass spectrometric analysis and were calculated to be 10 ± 3% and 17 ± 6%, respectively. The presence of dMn_3_O_4_ was clearly visible in the FE-TEM images of PTC-M (Fig. 3A), which was further confirmed by EDS analysis of PTC-M, showing Mn from Mn_3_O_4_, sulfur and carbon from SH-C12, and carbon, nitrogen, and oxygen from PEG (Additional file 1: Figure S5). Thermogravimetric (TGA) analysis was performed and the dMn_3_O_4_ loading was calculated to be 12% (Additional file 1: Figure S6B). ROS-non-sensitive micelles loaded with dMn_3_O_4_ (PC-M) were also prepared as controls by the same method.

To confirm the role of TK linkage in the ROS-responsive destabilization of PTC-M and release of dMn_3_O_4_, PTC-M was exposed to ROS conditions by adding H_2_O_2_. As controls, we utilized the ROS-non-sensitive PC-M. Both the PTC-M and PC-M exhibited a spherical morphology with tightly packed dMn_3_O_4_ in the nanomicelles, but after H_2_O_2,_ treatment, the supra-assembly of the PTC-M was distorted, followed by the release of dMn_3_O_4_, which was then aggregated as shown in the FE-TEM images, whereas the PC-M showed stable structures even after exposure to H_2_O_2_ (Fig. 3B and Additional file 1: Figure S4B). The ROS susceptibility of PTC-M was further confirmed by comparing the changes in hydrodynamic diameter and zeta potential with the PC-M, as shown in Additional file 1: Figure S7. The hydrodynamic diameter and zeta potential of the ROS-non-sensitive PC-M showed no discernible difference after the addition of H_2_O_2_. However, the ROS-sensitive PTC-M showed reduced hydrodynamic sizes, probably because of the removal of PEG shielding via TK breakage, resulting in destabilization of the structure. After the addition of H_2_O_2_, the zeta potential reverted from a negative to positive charge, which could be because of the removal of PEG and exposure of stearamine. We further studied the size variation by checking the stability over time by tracking the hydrodynamic diameters in the presence or absence of H_2_O_2_ (Fig. 3C). After the addition of H_2_O_2_, the hydrodynamic diameters significantly decreased within 10 s with no further changes with time up to six days, whereas PTC-M alone showed stable sizes during the experiment, suggesting the good colloidal stability of the PTC-M. We also checked the stability in 10% FBS and obseved that the PTC-M showed good stabilty upto 4 days (Additional file 1: Figure S9). The hydrodynamic diameters and zeta potential variation were also confirmed with ROS-sensitive and non-sensitive empty nanomicelles (PTC and PC) (Additional file 1: Figure S8). All these comparative studies in the presence or absence of ROS clearly demonstrated the stability and ROS sensitivity of the prepared nanomiclles or final dMn_3_O_4_ PTC-M nanozyme.

We further checked the H_2_O_2_ elimination property of the final PTC-M nanozyme due to dMn_3_O_4_ NP-loading. This was tested using terephthalic acid (TA) as a fluorescent probe. TA reacted with H_2_O_2_ to form 2-hydroxyterephthalic acid with a fluorescent peak at 425 nm. dMn_3_O_4_ will convert H_2_O_2_ to water and oxygen and hence, the fluorescent intensity decreases, confirming catalase-like activity, as shown in Fig. 3D. The PTC-M showed a strong reduction in fluorescence intensity compared to the empty micelle PTC in the presence of H_2_O_2_ over time. The catalase-like activity of PTC-M was also concentration-dependent, as shown in Additional file 1: Figure S10. The oxygen production capacity of PTC-M was further confirmed using an O_2_-specific fluorescent probe Ru(ddp)in the presence of H_2_O_2_. The fluorescence intensity of Ru(ddp)decreases in the presence of O_2_, suggesting the efficient production of O_2_. As shown in Fig. 3E, the fluorescence intensity of Ru(ddp) decreased in the presence of H_2_O_2_ in the PTC-M group, whereas the presence of H_2_O_2_ in the PTC-alone group showed little decrease in fluorescence (Fig. 3F), confirming the catalase activity resulting in oxygen production in PTC-M. Finally, we confirmed the H_2_O_2_ elimination property of PTC-M using an oxygen detection meter. As shown in Fig. 3G, the oxygen production by PTC-M was increased within the initial time, followed by a slow increase whereas the empty micelles PTC did not show oxygen production. The disproportionation of H_2_O_2_ was observed by the formation of oxygen bubbles in the PTC-M group as shown in Additional file 1: Figure S11A. It was also observed that PTC-M showed a higher production of oxygen compared to dMn_3_O_4_ alone, probably because of the close packing of dMn_3_O_4_ in PTC-M, making it more available for the reaction compared to free dMn_3_O_4_ (Additional file 1: Figure S11B). Even though the nanomicelles had loosened up due to H_2_O_2_ treatment, as shown in FE-TEM, dMn_3_O_4_ may still have been present inside the PTC-M, resulting in high catalase activity compared to free dMn_3_O_4_. All these results together confirmed the superior catalase-mimicking activity of the PTC-M.

### In vitro cell viability and cellular uptake

**Fig. 4:**
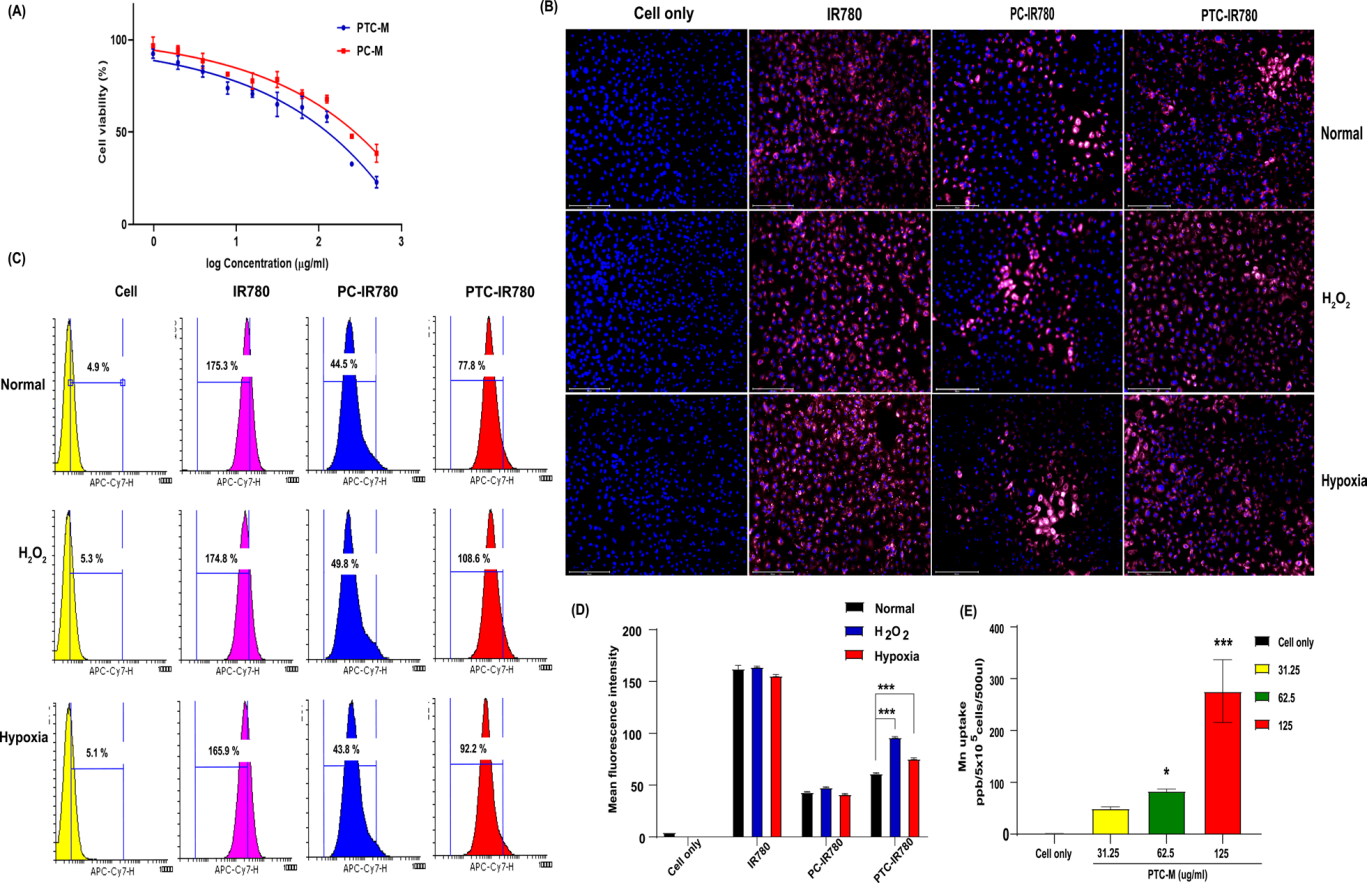
In vitro studies **A** IC50 analysis of PTC-M and PC-M, **B** Cellular uptake and intracellular distribution determined by fluorescence microscopy (scale bar = 200 µm), **C** Flow cytometry analysis and **D** Mean fluorescence intensity quantification of cellular uptake. **E** Cellular uptake of PTC-M determined by ICP-MS. Data is shown as mean ± SEM (n = 3 replicates). Statistics were performed by a one-way ANOVA (*P < 0.05, **P < 0.01, *** P < 0.001, and **** P < 0.0001)

To confirm the cellular uptake and, ROS susceptibility of intracellularly distributed ROS-sensitive-PTC and the release pattern of the payload, we compared PTC with PC in in vitro conditions. First, we checked the cell viability of the prepared PTC, PC, PTC-M, and PC-M in HK-2 cells by the WST-1 assay (Additional file 1: Figure S12). The empty micelles underwent little cell death, but after dMn_3_O_4_-loading, the cell viability decreased but the change was not statistically significant. Since the empty micelles were not cytotoxic, the slight variation in cell viability could be because of the surface-adsorbed dMn_3_O_4_. The IC50 value of PTC-M and PC-M was determined to be 130.41 and 247.17 ug/ml, respectively (Fig. 4A). To further examine the intracellular uptake and distribution of the nanomicelles, IR780 was used as a model drug and prepared IR780 loaded PTC nanomicells, PTC-IR780 to visualize the fluorescence intensity of IR780 using fluorescence microscopy (Evos FL Auto 2, Thermo Fisher Scientific, USA) (Fig. 4B). As a control group, we also analyzed the uptake of ROS-non-sensitive PC-IR780. Compared to PC-IR780, PTC-IR780 showed a higher uptake in HK-2 cells (Additional file 1: Figure S13). The exact reason behind the enhanced uptake of PTC-IR780 in non ROS condition is not clear, however it could be because the PTC could show slight sensitivity to the normal ROS level in cell culture conditions leading to PEG de-shielding leaving the surface charge slightly positive. PC-IR780 showed a similar uptake pattern regardless of ROS induced by H_2_O_2_ treatment or hypoxia-induced ROS conditions because of the non sensitive nature whereas, PTC-IR780 showed a different distribution pattern inside the cells, which could be due to the enhanced release of IR780 inside the cells. The previous cell viability data (Additional file 1: Figure S11) showing increased cell death in PTC-M would be also because of the enhanced cell uptake of PTC compared to PC. IR780 iodide, which is a highly hydrophobic dye that can show high affinity towards the cell membrane and showed a very bright signal regardless of the ROS exposure. The cellular uptake was further analyzed by checking the mean fluorescence intensity (MFI) by fluorescence-activated cell sorting (FACS) analysis (Fig. 4C), which supported the data obtained by fluorescence microscopy. As shown, the MFI was highest in the ROS-sensitive PTC-IR780 group compared to the other groups, confirming the enhanced release of IR780 in cells. We further quantified the MFI from various groups (Fig. 4D) which supported the microscopy data suggesting that PC-IR780 did not exhibit any significant difference in cellular uptake even under ROS induced conditions whereas, the PTC-IR780 showed a significant increase in cellular uptake when treated under ROS induced conditions. The cellular uptake of PTC was also determined by checking residual Mn in the cells after treatment with PTC-M. As shown in Fig. 4E, PTC-M showed a concentration-dependent uptake in the HK-2 cells. These results thus summarizes the enhanced internalization of nanomicelles and accelerated release inside the cells probably due to the ROS mediated disassembly of nanomicelles.

### Effect of PTC-M nanozyme on inflammation and apoptosis in hydrogen peroxide-stimulated HK-2 cells

**Fig. 5:**
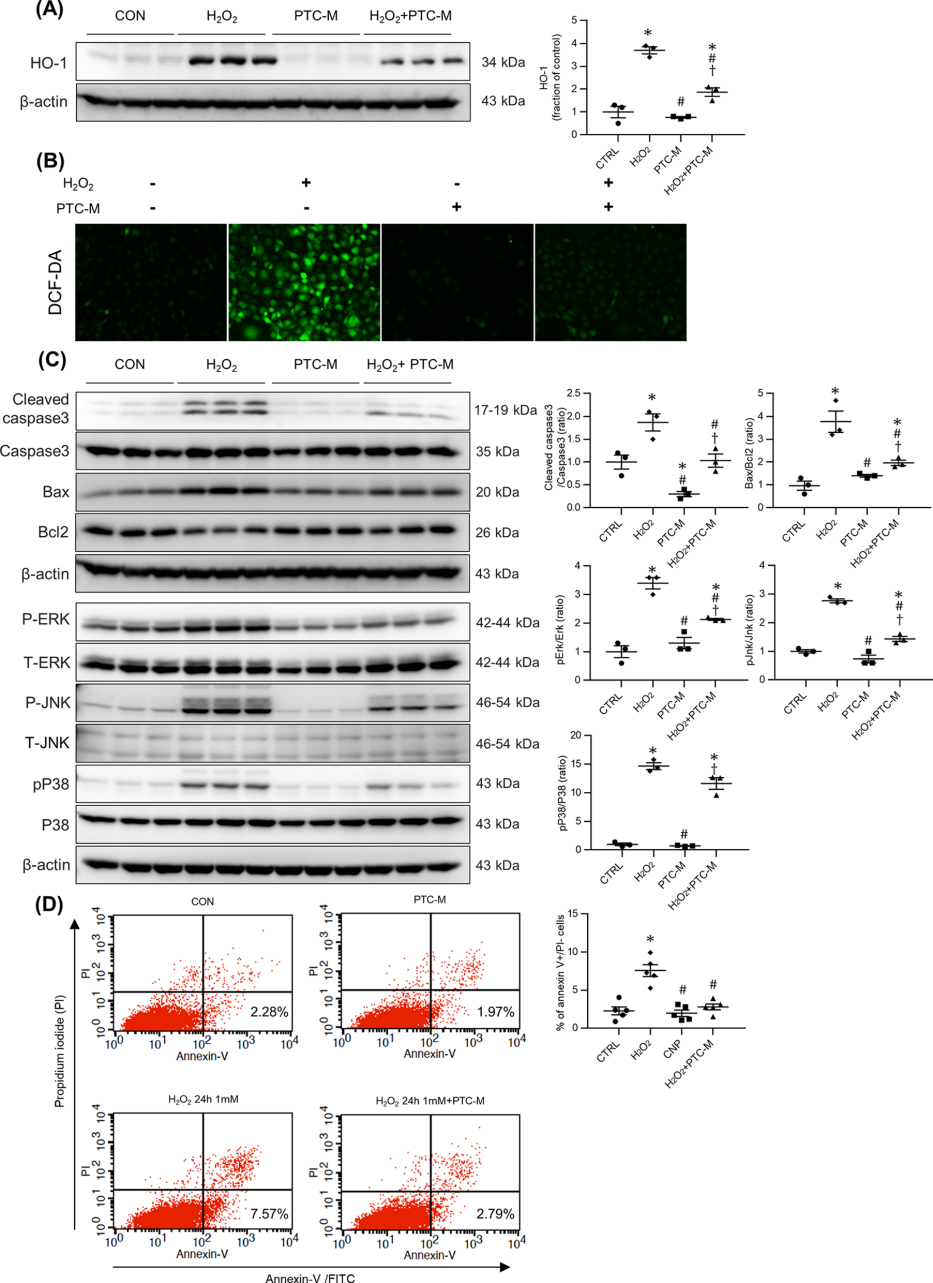
Effects of PTC-M on inflammation, apoptosis and MAPK pathway in H_2_O_2_ treated HK-2 cell. **A** Comparison of expression level for HO-1 proteins determined by immunoblotting in HK-2 after stimulation with vehicles or H_2_O_2_. β-actin was used as the endogenous control. **B** Images of the effects of H2O2-induced ROS production by PTC-M imaged by fluorescence microscope. Cytosolic ROS was labeled using DCF-DA probes. **C** Comparison of expression level for apoptosis and MAPK proteins determined by immunoblotting in HK-2 after stimulation with vehicles or H_2_O_2_. **D** Flow cytometry results with Annexin V-FITC/PI staining. Apoptotic cells were defined as Annexin V^+^ and PI^−^. Data are shown as mean ± SEM. *P < 0.05 vs. control (CON) group; ^#^P < 0.05 vs. H_2_O_2_. group; ^†^P < 0.05 vs. PTC-M group.* HO-1* heme oxygenase-1, *DCF-DA* 2′,7′-dichlorodihydrofluorescein diacetate, *MAPK* mitogen-activated protein kinase

We conducted in vitro studies in HK-2 cells to further explore the effects of PTC-M on oxidative stress conditions at the cellular level. HK-2 cells were stimulated with H_2_O_2_, a representative ROS (Fig. 5A). H_2_O_2_ enhanced heme oxygenase (HO)-1 expression, which was ameliorated by PTC-M co-treatment (Fig. 5A). The 2′,7′-dichlorodihydrofluorescein diacetate (DCF-DA) assay was performed to check the pattern of intracellular ROS change. The HK-2 cells stimulated by H_2_O_2_ showed high fluorescence intensity, but after co-treatment with PTC-M, the fluorescence intensity was decreased (Fig. 5B). The BAX/BCL-2 ratio was increased by H_2_O_2_ treatment, which was also recovered by PTC-M cotreatment, indicating protection against apoptosis (Fig. 5C). HK-2 cells treated with H_2_O_2_ exhibited a significant progressive increase in annexin V^+^/PI^−^ staining, which was prevented by PTC-M (Fig. 5D). H_2_O_2_-induced ERK, JNK, and p38 phosphorylation was also suppressed by co-treatment with PTC-M, although a significant reduction in phosphorylated P38 was not observed (Fig. 5C). These data suggest that PTC-M suppressed ROS-induced apoptotic processes in renal tubular epithelial cells.

## Biodistribution of PTC-M nanozyme in IRI mice

**Fig. 6:**
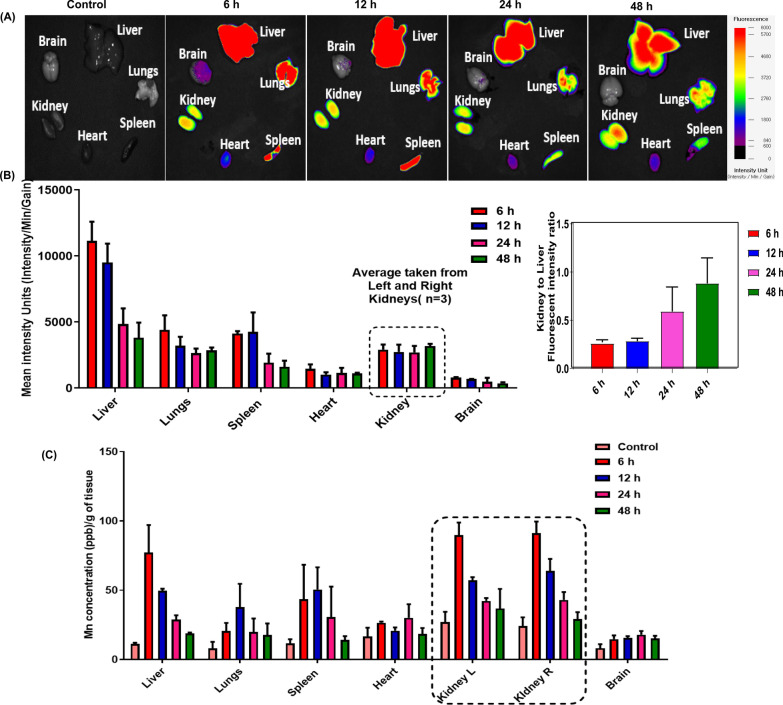
In vivo biodistribution **A** Ex vivo images of major organs at different time point post injection (n = 3). **B** NIRF signal intensity of the organs and kidney to liver ratios at different time point, **C** Mn concentrations in different organs at different time points

The biodistribution of PTC was investigated in IRI mice by loading the fluorescent dye IR780 as a model drug (PTC-IR780). We also compared the biodistribution of the PC-IR780 and IR780 alone and observed that all groups showed fluorescence in major organs however PTC-IR780 showed an enhanced accumulation in kidney unlike PC-IR780 or IR780 group (Additional file 1: Figure S14 A). We also checked the biodistribution profile of PTC-IR780 in normal mice and found that it showed higher liver accumulation which is common for the nanoparticle after intravenous injection but has not accumulated much in kidneys (Additional file 1: Figure S15). Thus, we conclude that the enhanced kidney accumulation of PTC is predominantly mediated by kidney inflammation. In our study, we designed PTC nanomicelles in such a way that it circulates in blood and gradually accumulates at the inflammation site and after reaching the inflammation site, the PTC gets destabilized by the presence of enhanced ROS and the loaded IR780 will be released which stays in the kidney for a longer time resulting in enhanced fluorescence. The kidney accumulation among all groups were compared by quantifying the ex-vivo fluorescence intensity and observed that PTC showed a superior kidney accumulation even though it shows fluorescence in other major organs which is inevitable in case of systemic injection (Additional file 1: Figure S14 B) As shown in Fig. 6A, strong IR780 fluorescence intensity was observed in the kidneys from 6 h post-injection of PTC-IR780. The fluorescence intensity of the main organs was high in the order of liver, lungs, spleen, and kidneys. The fluorescence intensity of other organs decreased over time, while the fluorescence intensity of the kidneys remained relatively constant until 48 h. Thus, the fluorescence intensity of the kidneys versus the liver tended to increase gradually over time (Fig. 6B). This could be consistent with previous studies reporting that an increase in kidney inflammation could lead to enhanced permeability of the vessels and hence, increased accumulation of NMs in the kidneys [16]. The biodistribution was further confirmed by checking the Mn content in different organs using inductive-coupled plasma mass spectrometer (ICP-MS) analysis. As shown in Fig. 6C, the Mn concentration in both kidneys peaked at 6 h post-injection and gradually declined until 48 h. When comparing the tissue Mn concentration with that of the other main organs, the kidneys reached a higher tissue Mn concentration than the liver, lungs, and spleen at any time point. To confirm the clearance of the PTC-M, we have performed the ICP-MS analysis of all organs, feces, and urine at the end of the study and after a week and we observed that major portion of the Mn was shown in intestine and feces. It was observed that the Mn clearence primarily took a hepatobiliary route rather than renal clearance. We assume that after accumulating in kidney the Mn_3_O_4_ nanoparticle gets degraded to Mn ions which follows the usual hepatobiliary clearence of Mn (Additional file 1: Figure S16)[17].

### PTC-M nanozyme decreases kidney damage marker and attenuates morphological changes in IRI mice kidneys

**Fig. 7:**
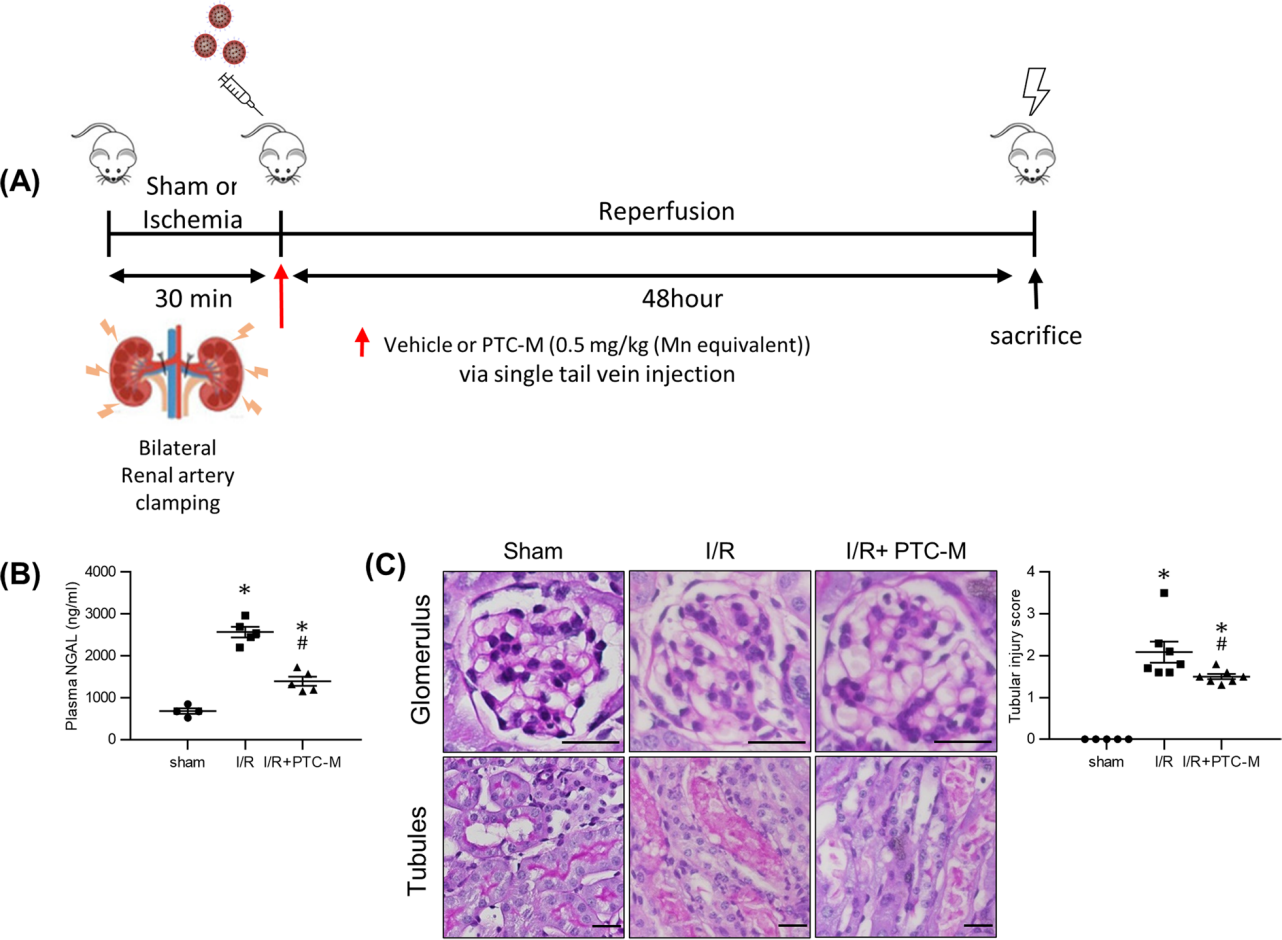
Effects of PTC-M on kidney damage marker and morphological changes in IRI mice kidneys. **A** A schematic plot of the timeline of ischemia–reperfusion injury (IRI), PTC-M treatment and experimental analyses. **B** Plasma neutrophil gelatinase-associated lipocalin (NGAL) level of each mice group. **C** Periodic Acid-Schiff staining of kidney sections from sham, IRI mice or IRI mice treated with PTC-M. Scale bars, 25 μm. Data are shown as mean ± SEM. *P < 0.05 vs. sham mice; ^#^P < 0.05 vs. IRI mice

Finally, we performed an in vivo experiment to determine the efficacy of PTC-M to decrease renal damage in renal IRI. To investigate the overall status of damage in IRI kidneys, we measured plasma neutrophil gelatinase-associated lipocalin (NGAL), a well-known marker of tubular injury in AKI [18]. The level of plasma NGAL was significantly higher in the IRI group than in the sham group but was reduced by PTC-M treatment (Fig. 7B). We conducted periodic acid-Schiff (PAS) staining of the kidney sections to check for morphological changes (Fig. 7C). In contrast to the sham mice, renal tubular swelling, and dilatation filled with necrotic material were observed. Inflammatory cells infiltrating the interstitial spaces were observed in IRI mice kidneys. These changes were improved by PTC-M treatment. Tubular swelling, necrotic materials in the tubules, and inflammatory cell infiltration were relatively attenuated in the PTC-M-treated IRI mice. Unlike changes in the tubules, there were no significant morphological differences observed in the glomeruli in each group.

### PTC-M ameliorates inflammation and apoptosis in IRI mice kidneys

**Fig. 8:**
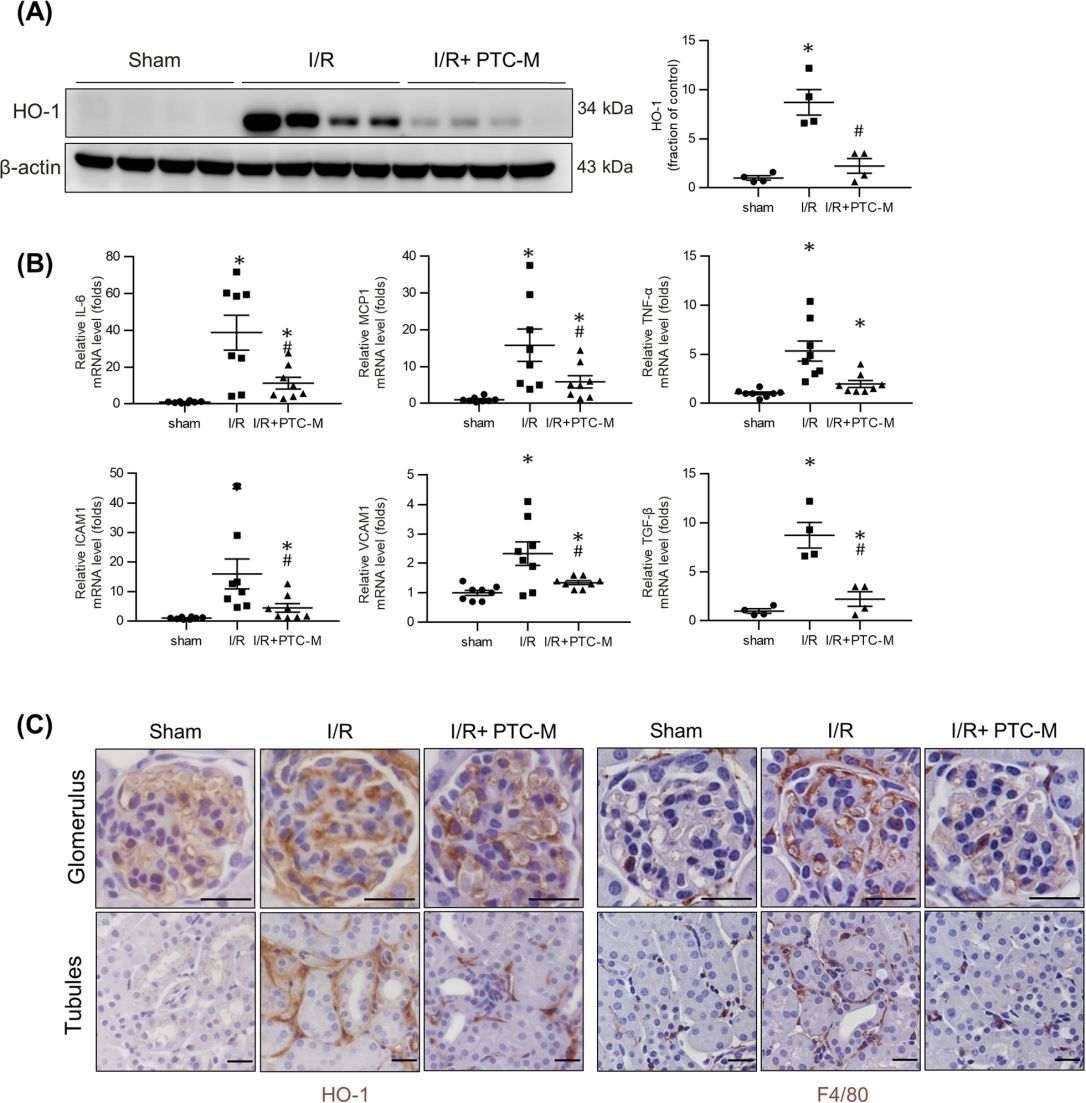
Effects of PTC-M on inflammation in IRI mice kidneys. **A** Comparison of protein expression level for HO-1 determined by immunoblotting from the kidney of sham, *IRI,* and IRI + PTC-M mice. β-actin was used as the endogenous control. **B** Comparison of mRNA expression level for inflammatory markers determined by qPCR from the kidney of sham, IRI, and IRI + PTC-M mice. **C** Representative images of immunohistochemical staining for HO-1 (left) and F4/80 (right) in the kidney of sham, IRI and IRI + PTC-M mice. Scale bars, 25 μm. Data are shown as mean ± SEM. *P < 0.05 vs. sham mice; ^#^P < 0.05 vs. IRI mice. *MCP-1* monocyte chemoattractant protein-1, *TNF-α* tumor necrosis factor-α, *ICAM-1* intercellular adhesion molecule-1, *VCAM-1* vascular cell adhesion molecule-1, *TGF-β* transforming growth factor beta

To examine whether the PTC-M could reduce the inflammation induced by the oxidative injury, we examined the protein expression level of HO-1 by western blot analysis to assess the degree of oxidative stress in IRI mice kidneys. We found considerably elevated expression levels of HO-1 in the kidneys of IRI mice, which were attenuated by PTC-M treatment (Fig. 8A). Immunohistochemical staining for HO-1 showed its increased expression in the mesangium of the glomeruli, periglomerular spaces, and peritubular interstitial space of IRI mice kidneys, which was reduced in PTC-M-treated mice kidneys (Fig. 8C). To elucidate the active inflammation in the kidneys of IRI mice, we checked the transcript levels of proinflammatory cytokines and adhesion molecules by quantitative polymerase chain reaction (qPCR) analysis (Fig. 8B). The mRNA expression levels of interleukin (IL)-6, monocyte chemoattractant protein (MCP)-1, tumor necrosis factor (TNF)-α, intracellular adhesion molecule (ICAM)-1, vascular cell adhesion molecule (VCAM-1), and transforming growth factor (TGF)-β were significantly increased in the kidneys of IRI mice, and the treatment with PTC-M suppressed these changes (Fig. 8B). Immunohistochemical staining for F4/80, a marker of murine macrophages, revealed increased expression in the periglomerular and interstitial spaces of the kidneys of IRI mice, which was also attenuated by PTC-M treatment (Fig. 8C). These data suggest that PTC-M suppressed renal inflammation and oxidative stress in IRI mice.

**Fig. 9:**
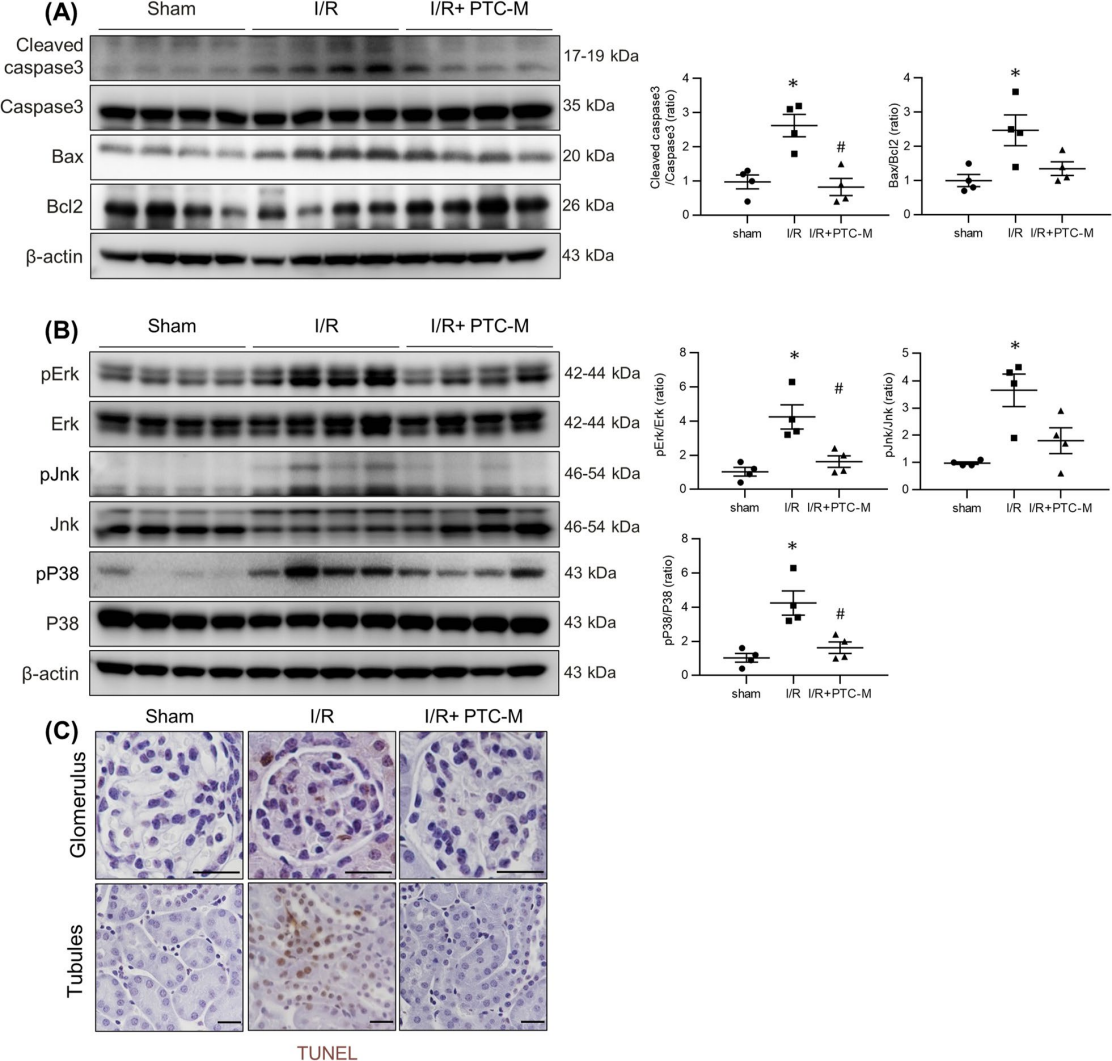
Effects of PTC-M on apoptosis and MAPK pathway in IRI mice kidneys. **A** Comparison of protein expression level for proteins related to apoptosis determined by immunoblotting from the kidney of sham, IRI, and IRI + PTC-M mice. **B** Protein expression of the total ERK, phosphorylated ERK (p-ERK), total JNK, phosphorylated JNK (p-JNK), P38 and phosphorylated P38 (pP38) was assessed in the kidney of sham, IRI, and IRI + PTC-M mice. β-actin was used as the endogenous control. **C** Representative images of TUNEL staining in the kidney of sham, IRI and IRI + PTC-M mice. Scale bars, 25 μm. Data are shown as mean ± SEM. *P < 0.05 vs. sham mice; ^#^P < 0.05 vs. IRI mice. *TUNEL *Terminal deoxynucleotidyl transferase dUDP nick-end labeling

To evaluate the degree of renal tubular cell death in IRI mice, protein expression level of apoptotic proteins was assessed. Immunoblotting revealed an increased Bax/Bcl-2 ratio and cleaved caspase 3/caspase 3 ratio in the kidney tissue of IRI mice, suggesting that apoptosis was exacerbated (Fig. 9A). These changes were not observed in the mice treated with PTC-M. PTC-M treatment significantly reduced apoptotic cascades in kidney of IRI mice induced by oxidative stress. To determine whether PTC-M inhibited ROS-mediated apoptosis via mitogen-activated protein kinase (MAPK), we estimated changes in the MAPK signaling pathway. We performed immunoblot analysis for MAPK pathway protein level and their phosphorylated (activated) forms (Fig. 9B). The levels of phosphorylated ERK, JNK, and P38 were enhanced in the kidneys of IRI mice compared to the sham mice, which were attenuated by PTC-M, although a significant reduction in phosphorylated JNK was not observed. Terminal deoxynucleotidyl transferase dUDP nick-end labeling (TUNEL) staining demonstrated more TUNEL-positive tubular epithelial cells in the cortex of the kidneys of IRI mice compared to that of sham mice, whereas PTC-M treatment decreased the numbers of TUNEL-positive cells (Fig. 9C). These results suggest that PTC-M reduced apoptosis in IRI mice kidneys by affecting the MAPK signaling pathway. These findings in IRI mouse kidneys were consistent with the results in H_2_O_2_-stimulated human kidney cells, demonstrating a common mechanism of PTC-M renal protection. H&E staining was performed on major organs collected from normal and PTC-M treated group and no major histological changes indicating damages were observed (Additional file 1: Figure S17).

## Discussion

The results of present study revealed that PTC-M could ameliorate the injury of kidney in IRI mice as representative experimental model of AKI. Especially, PTC-M significantly reduced inflammation and apoptosis in the kidneys of IRI mice and protected against H_2_O_2_-induced cellular injury in human proximal tubular epithelial cells. These findings highlight the potential of PTC-M nanozyme as a candidate for therapeutic agent not only for IRI but also for AKI due to other causes.

Mn_3_O_4_ nanoparticles possess double-oxidation state Mn^2+^ and Mn^3+^ and protect the cells from ROS-mediated damage by preventing oxidative damage of the cellular components during high oxidative stress conditions. Apart from these benefits, the synthesis procedure of Mn_3_O_4_ is simple, where even a weak base can induce its formation [19–21]. Despite the great promise, Mn_3_O_4_-based nanoparticles have not been exploited much for in vivo applications. Previously, the ROS-scavenging property of Mn_3_O_4_ nanoparticles was utilized for cytoprotection [22], for developing bio-semiconductors [20], and for relieving oxidative stress in plants [23]. However, the small particle size and the instability or possible agglomeration of these nanoparticles can adversely affect the proper distribution and circulation half-life when used in in vivo conditions. Since ordinary Mn_3_O_4_ cannot be targeted, the potential toxicity to unwanted sites due to higher dosages could also be a safety concern in in vivo conditions. Hydrophobically modified Mn_3_O_4_ NPs can ease this situation by allowing them to get loaded into a nanocarrier, thereby endowing it with the ability to passively target the site of action. Here, we synthesized 1-dodecanethiol stabilized hydrophobic Mn_3_O_4_ nanoparticles (dMn_3_O_4_ NPs) and utilized their catalase-like activity to reduce kidney damage from IRI.

Recently, TK linkers have gained attention in developing ROS-responsive delivery systems to ROS-rich inflammation sites. TK bonds will be cleaved instantly by ROS oxidants, resulting in the loss of structural integrity of nanoparticulate systems, accelerating the release of loaded therapeutic agents [24–26]. Moreover, TK bonds are stable under physiological conditions. In this work, we utilized an ROS-sensitive amphiphilic polymer PEG-stearamine conjugate developed by hydrophilic PEG connected to hydrophobic C18 alkyl chains via a TK linker (PEG-TK-C18, PTC) previously synthesized by our group [15]. ROS-sensitive nanomicelles were constructed by the self-assembly of PTC and the prepared dMn_3_O_4_ NPs were loaded inside, resulting in a versatile nanoplatform-ROS-sensitive nanozyme, PTC-M. The prepared PTC-M nanozyme contained different domains (as shown in Fig. 1): (1) the outer PEG shell endowed biocompatibility, dispersity, and prolonged circulation half-life in vivo and selective accumulation in inflammatory sites via extravasation through leaky vasculature and subsequent inflammatory cell-mediated sequestration (ELVIS) [27], (2) the TK linker, which is cleavable in an ROS-rich environment, (3) the C18 segment offering a hydrophobic core for incorporating dMn_3_O_4_ NPs as well as enhanced cell uptake, and (4) dMn_3_O_4_ NPs for ROS elimination. The other advantages included the simple synthesis procedure of dMn_3_O_4_ NPs. After administration, PTC-M circulated in the blood and accumulated at the ROS-rich inflammatory sites, followed by the instantaneous dissociation of TK linkage by ROS and destabilization of PTC-M, enabling the stepwise release and activation of the dMn_3_O_4_ NPs.

The characteristics of renal IRI are active inflammation by immune responses to ischemic tissue injury and elevated oxidative stress. Reduced tissue perfusion leads to acute tubular injury, which triggers inflammatory consequences and induces tubular epithelial cell death. These damaged cells amplify the inflammatory response again and create a vicious cycle. It is well known that the balance between pro-inflammatory and anti-inflammatory mediators determines tissue injury or repair [28]. Our study showed the upregulation of HO-1 in IRI mice kidneys, which was suppressed by PTC-M. HO-1, known as an endogenous anti-inflammatory enzyme, modulates immune responses following ischemia [29, 30]. The increased expression of HO-1 represents anti-inflammatory and antioxidative responses against IRI. The reduction in HO-1 expression by the administration of PTC-M indirectly implied a decrease in the inflammatory response in the tissues. In contrast, IL-6, MCP-1, and TNF-α are known as pro-inflammatory mediators and play roles in exacerbating tissue inflammation. ICAM-1 and VCAM-1 contribute to worsening inflammation by promoting the recruitment of inflammatory cells. PTC-M also reduced the expression of these molecules. Since these inflammatory cytokines trigger the systemic inflammation cascade, it is very important to suppress the inflammatory response to damaged kidneys.

The reestablishment of renal perfusion activates vascular endothelial cells, which triggers not only the release of inflammatory cytokines but also the production of ROS [31]. Activated neutrophils also produce and release ROS through the activation of NADPH oxidase 2 by proinflammatory cytokines [32]. Excessive ROS causes damage to cellular proteins, lipids, nucleic acid, membranes, and mitochondria. The accumulation of intracellular ROS leads to oxidative stress conditions and the activation of cell death pathways. Thus, a reduction in tubular cell apoptosis is required for the treatment of renal IRI. The results of our study showed that the expression of the pro-apoptotic protein Bax was increased, and the expression of the anti-apoptotic protein Bcl-2 was decreased, resulting in an increase in the Bax/Bcl-2 ratio, consistent with previous studies [33]. PTC-M effectively attenuated IR-induced apoptosis, represented by a decrease in the Bax/Bcl-2 ratio. Cleaved caspase 3, an executioner caspase activated in both the extrinsic and intrinsic pathways, was also reduced by PTC-M treatment. The MAPK pathway is known to be involved in diverse cellular responses for survival against external stimuli. These responses include cellular proliferation, differentiation, inflammation, and apoptosis [34]. In previous studies, MAPK signaling pathways were activated after IR injury [35, 36]. In our results, the phosphorylation of ERK, JNK, and p38 MAPK was increased after IR injury and ameliorated by PTC-M treatment in both in vivo and in vitro experiments.

## Conclusion

In summary, ROS-sensitive nanozyme PTC-M composed of hydrophobic dMn_3_O_4_ were successfully prepared and evaluated. PTC-M could passively target the kidneys and release dMn_3_O_4_ by responding to high levels of ROS present at the site, and the released dMn_3_O_4_ could scavenge the excessive ROS more effectively. Thus, PTC-M can overcome the shortcomings of dMn_3_O_4_ by increasing its solubility, stability, and biocompatibility, and can reduce the dose of dMn_3_O_4_. It can also synergistically exert anti-inflammatory and anti-apoptotic effects. The present results demonstrated that PTC-M could ameliorate renal injury in IRI mice as an experimental model of AKI. Specifically, PTC-M effectively attenuated inflammation and apoptosis in the kidneys of IRI mice and protected against H_2_O_2_-induced cellular injury in human proximal tubular epithelial cells. These findings highlight the potential of PTC-M as a therapeutic agent not only for IRI but also for AKI.

## Materials and methods

### Materials

Sodium hydroxide (NaOH), 1-Dodecanethiol, stearamine (C18), 1-ethyl-3-(3-dimethylaminopropyl) carbodiimide (EDC), N-hydroxysuccinimide (NHS), and 3-mercaptopropionic acid and triethylamine (TEA) were procured from Sigma-Aldrich (St. Louis, MO, USA). PEG-AM (2 KDa) was purchased from PEG-Shop (SunBio Inc., South Korea). Manganese (II) chloride dihydrate (MnCl_2_.2H_2_O) and N, N-dimethylformamide (DMF) were purchased from Merck (Darmstadt, Germany). Tris (4 7-diphenyl-1 10-phenanthroline) ruthenium(ii) dichloride (Ru(ddp)) was purchased from chemcruz (Santa Cruz Biotechnology, Texas, USA) Other solvents and reagents were of analytical grade and used as received.

### Synthesis and characterization of dMn_3_O_4_ nanoparticles

dMn_3_O_4_ nanoparticles were prepared as follows. Briefly, 4 mmol NaOH in 2.5 ml of distilled water (DW) was added dropwise to 2 mmol MnCl_2_ dissolved in 2.5 ml of DW under strong stirring with the solution turning brown. Later, 1 mmol of 1-dodecanethiol was added to the mixture during constant stirring till the solution became a paste. The precipitate was washed in ethanol by centrifugation at 5000 rpm for 5 min. Washing was repeated five times and the final pellet was dried, washed in DW twice, and lyophilized.

The morphology of the prepared dMn_3_O_4_ NPs was analyzed by FE-TEM, (JEM-2100F; JEOL, Tokyo, Japan) operated at 200 kV. To investigate the state of Mn in dMn_3_O_4_, XPS analysis was performed using a k-alpha X-ray photoelectron spectrophotometer system (Thermo Fisher Scientific, Waltham, MA, USA). XRD measurements were performed using X-ray diffraction, M18XHF-SRA (Macscience, Japan), and the XRD pattern was analyzed using the ICDD (International centre for Diffraction Data) file.

### Synthesis and characterization of PTC and PC

The synthesis of TK linker, TK linker-conjugated polyethylene glycol (PEG-TK), and (PEG)-stearamine (C18) conjugate (PTC) was performed according to our previous publication [15]. As a control, we also prepared ROS-non-sensitive polymer (PC) by conjugating stearic acid to PEG-AM. Briefly, 200 mg of m-PEG amine was reacted with 160 mg of stearic acid with 160 µl of TEA, 480 mg of EDC, and 290 mg of NHS in 20 ml of DMF at 37 ℃ overnight with stirring under N_2_ purging. The sample was then purified by dialysis (MWCO = 12–14 KDa) against DW for one day, followed by freeze-drying. The chemical structures of the synthesized PTC and PC were confirmed using proton nuclear magnetic resonance (^1^H NMR) spectroscopy (400 Hz, Bruker: Billerica, MA, USA) in chloroform (CHCl_3_). ROS-sensitive PTC and ROS-non-sensitive PC empty micelles were prepared by the self-assembly of PTC and PC under sonication and dialysis.

### Preparation and characterization of ROS sensitive nanozyme (PTC-M)

PTC-M was prepared by the dialysis method. Briefly, 2 mg of dMn_3_O_4_ and 10 mg of PTC were dissolved in 1 mL of anhydrous dimethyl sulfoxide (DMSO). The mixture was then added dropwise to 20 ml of DW under probe sonication (30% amplitude, 5 s ON/OFF, 5 min). The solution was then dialyzed (MWCO = 12–14 KDa) against DW for one day, followed by freeze-drying. Similarly, dMn_3_O_4_ NP-loaded ROS-non-sensitive nanomicelles (PC-M) were also prepared as a control group. The hydrodynamic diameters and zeta potential of the prepared empty nanomicelles (PTC and PC) and dMn_3_O_4_ NP-loaded nanomicelles (PTC-M and PC-M) were evaluated by dynamic light scattering (Zetasizer Nano Z instrument, Malvern, UK). The morphology of the prepared PTC and PTC-M were visualized using FE − TEM. The ROS-sensitive destabilization via the degradation of the TK linker of PTC-M was observed by DLS and FE-TEM analysis after treating the samples with 1 mM H_2_O_2_. The loading content of dMn_3_O_4_ in the final PTC-M was analyzed using a thermogravimetric analyzer (TGA N-1000, Scinco, Seoul, Korea) and an ICP-MS (820 ICP-MS Varian Bruker, Billerica, MA, USA) analysis.

The catalase-like activity of the final PTC-M was evaluated by observing the catalytic elimination of H_2_O_2_ by a fluorescent method using TA. Briefly, 1 mM H_2_O_2_ was added to the samples, followed by the addition of 0.5 mM TA (DMF). The samples were vortexed, and the fluorescence was recorded (excitation: 320 nm; emission: 425 nm) for up to 45 min. The catalase-like activity of PTC-M at different concentrations was also checked by a similar method.

H_2_O_2_ decomposition to water and oxygen was also confirmed using Ru(ddp) as a probe for oxygen generation. Briefly, 1 mM H_2_O_2_ was added to the samples, followed by the addition of 1 µM Ru(ddp) _(_DMF). The samples were mixed thoroughly and the fluorescence intensity at excitation 440 nm and emission 530 – 730 nm was recorded. Later, oxygen production was also confirmed by determining the dissolved oxygen concentration using a dissolved oxygen sensor (RDO Optical Dissolved Sensor, Thermo Scientific).

### Cell culture and reagents

The in vitro studies were conducted as previously described [37]. In short, human renal proximal tubular epithelial cells (HK-2 cells, American Type Culture Collection, Manassas, VA, USA) were used for the in vitro studies. HK-2 cells were cultured and passaged every 3 – 4 days in 100-mm dishes containing a combination of Dulbecco’s modified Eagle’s (DMEM) and Hams F-12 medium (Welgene, Daegu, Korea) supplemented with 10% fetal bovine serum (FBS; Welgene), 100 U/ml penicillin, and 100 mg/ml streptomycin (Sigma-Aldrich). The HK-2 cells were then incubated in a humidified atmosphere of 5% CO_2_ and 95% air at 37 °C for 24 h, and sub-cultured until 70 – 80% confluent. The HK-2 cells were plated onto 60-mm dishes in medium containing 10% FBS and incubated for 24 h. The cells were then incubated in DMEM-F12 medium without FBS and treated with H_2_O_2_ (1 mM) for an additional 24 h (except, 15 min for ERK, JNK, and p38). PTC-M (2 ug) was added 1 h prior to H_2_O_2_ treatment. All the in vitro experiments were repeated twice to determine reproducibility and done within the 30th passage of the cells.

### Cell viability

In vitro cell viability was evaluated in HK-2 cells (1 × 10^4^ cells/well) in DMEM F12 medium incubated overnight. The media was aspirated, and cells were incubated with different concentrations of PTC and PTC-M for 24 h followed by the WST-1 assay, performed according to the manufacturer’s protocol. To compare the influence of TK linkages, we also compared the viability of cells treated with PC and PC-M. The IC50 values of the samples were analyzed from the cell viability data. Non-treated cells were used as the positive controls and cells treated with 0.1%Triton × 100 were used as the negative controls.

### Cellular uptake

To investigate the intracellular distribution and release of payload from PTC, PTC loaded with a near-infrared (NIR) dye, IR780 iodide (PTC-IR780), was developed. For the uptake studies, HK-2 cells (3 × 10^4^ cells/well) were seeded in Lab-Tek® Chamber Slides and incubated overnight. The media was aspirated, and different samples of IR780 only, PTC-IR780 and PC-IR780 with 4 ug/ml equivalent IR780 per well were added to the wells, followed by a 4-h incubation. Cells with no treatment were used as controls. After incubation, the media were aspirated, followed by DPBS washing and 4% paraformaldehyde (PFA) fixation. The cells were then counterstained with 4′,6-diamidino-2-phenylindole (DAPI). The fluorescence intensity of IR780 was analyzed by fluorescence microscopy (Evos FL Auto 2, Thermo Fisher Scientific) to determine IR780 uptake by the cells. To show the ROS mediated destabilization of the nanomicelles and release of the fluorescent dye as a model drug, an oxidative stress injured HK2 cell model was used. The oxidative stress was induced by H_2_O_2_ treatment,200 µM H_2_O_2_ for 2 h or by exposing the cells under hypoxic environment by incubating the cells in a hypoxia chamber (1% O_2_, 5% CO_2_) for 24 h prior to treatment. Cellular uptake was further quantified by flow cytometry analysis. Briefly, 1 × 10^5^ HK-2 cells/well were seeded in 12-well plates and incubated overnight. The media was aspirated, and different samples were added followed by 4 h incubation. The cells were then collected and centrifuged to obtain the pellet followed by two DPBS washes. The cells were then resuspended in FACS buffer containing 3% FBS and fluorescence-activated cell sorting (FACS) was performed to measure the uptake capability of each group. The data was gated with an APC-Cy 7 filter. Cellular uptake of the final PTC-M nanozyme was determined via ICP-MS analysis. Briefly, 1 × 10^5^ of HK-2 cells/well were seeded in 12-well plates followed by overnight incubation. The media was then removed, and different concentrations of PTC-M were added to the wells and incubated for another 4 h. After the incubation, the cells were collected and centrifuged to obtain the pellet, which was washed with DPBS twice. Aqua regia (1 ml) was added to the final pellet for digestion and the samples were then diluted and analyzed for Mn content by ICP-MS. Non-treated cells were used as the controls.

### Flow cytometry

Flow cytometry analysis was conducted as previously described [38]. An annexin V FLUOS staining kit (Sigma-Aldrich) was used to measure the level of annexin V binding according to the manufacturer’s instructions. Briefly, after treatment with 0 or 1 mM H_2_O_2_ for 24 h with or without 2 ug of PTC-M pretreatment, HK-2 cells were harvested and washed twice with pre-cooled phosphate-buffered saline and resuspended in a binding buffer containing annexin V. After incubation in the dark for 15 min, the cells were analyzed by flow cytometry (Becton–Dickinson, San Jose, CA, USA). Several controls were used to optimize the instrument settings and determine the gating for the Windows-based platform. Apoptotic cells were defined as PI-negative and Annexin V-FITC positive.

### Determination of intracellular ROS generation

To determine ROS generation, we used the fluoroprobe DCF-DA (Molecular Probes, Eugene, OR, USA) as previously described [38]. HK-2 cells were incubated with 0 or 1 mM H_2_O_2_ for 24 h with or without 2 ug of PTC-M pretreatment, and washed twice with Hank’s balanced salt solution, then incubated with Hank’s balanced salt solution (without phenol red) containing DCF-DA for 30 min at 37 °C in the dark. Images were obtained with a fluorescence microscope (Nikon, Tokyo, Japan).

### Experimental animals and protocols

All experimental methods were performed in accordance with the relevant guidelines and regulations. The experimental protocol was approved by the Animal Care Regulations Committee of Chonnam National University Hospital (CNUHIACUC-20009).

Seven-week-old male C57BL6 mice weighing 20 – 22 g were purchased from Samtako (Korea). The mice were housed at the animal care facility at Chonnam National University medical school and fed a standard diet with ad libitum access to water. The mice were divided into three groups: sham (n = 4), IRI (n = 4), and IRI with PTC-M treatment (n = 4). To induce the IRI mice model, surgery was performed as follows. After anesthesia induction using an intraperitoneal injection of ketamine (70 mg/kg; Yuhan, Seoul, South Korea) and xylazine (7 mg/kg; Bayer, Leverkusen, Germany), a midline incision was made to expose the abdominal cavity and both renal pedicles were clamped with a micro clip (ROBOZ, Gaithersburg, MD, USA) for 30 min on a temperature-regulated table (38.5 °C) to maintain body temperature. The sham group (n = 4) underwent the same procedure, except that the clip was not applied.

### In vivo biodistribution

For biodistribution analysis, the IRI group was injected intravenously with PTC-IR780 at a dose of 0.5 mg/kg (IR780 equivalent). The mice were euthanized at predetermined times (6, 12, 24, and 48 h) and the major organs were collected. Fluorescence signals from the collected organs were analyzed using the fluorescence-labeled organism bio-imaging instrument (FOBI, NEO science, Gyeonggi, Korea). The fluorescence intensity of the anatomized key organs was measured to evaluate the organ accumulation of PTC-IR780. Biodistribution was further compared with PC-IR780 or IR780 after administering equivalent amount of IR780. The PTC-IR780 biodistribution was also compared in normal mice after intravenous administration.

Biodistribution analysis of the final PTC-M samples was also performed. Briefly, the IRI group was injected with 0.5 mg/kg of the PTC-M (Mn equivalent) samples, and the mice were euthanized at the predetermined time intervals mentioned above and the major organs were collected. The organs were then treated with aqua regia, and the samples were analyzed using ICP-MS to determine the tissue Mn concentration.

To check the therapeutic effect of PTC-M in vivo, the treatment group was given a single dose of 0.5 mg/kg PTC-M via tail vein injection after surgery. The mice were euthanized after 48 h of reperfusion. Blood samples were collected from the heart, and the left kidney was rapidly removed and processed for Western blotting or fixed in 4% paraformaldehyde solution for immunohistochemistry (IHC). The right kidney was frozen at − 80 °C for real-time polymerase chain reaction (PCR) analysis.

### Plasma NGAL measurement

Plasma neutrophil gelatinase-associated lipocalin (NGAL) levels were measured with a commercial ELISA kit (R&D Systems, Minneapolis, MN, USA) according to the protocol provided by the manufacturer. A 1:400 sample dilution was performed for the plasma NGAL measurements.

### Semiquantitative immunoblotting

Western blot analysis was performed as previously described [37]. Kidney tissues were homogenized in ice-cold isolation solution containing 0.3 M sucrose, 25 mM imidazole, 1 mM ethylenediaminetetraacetic acid (EDTA), 8.5 mM leupeptin, and 1 mM phenylmethylsulfonyl fluoride (pH 7.2). The homogenates were centrifuged at 4000 × g for 15 min at 4 °C to remove whole cells, nuclei, and mitochondria. The total protein concentration was measured by a bicinchoninic acid (BCA) assay kit (Pierce; Rockford, IL, USA). All samples were adjusted to reach the same final protein concentrations. They were then dissolved at 65 °C for 15 min in sodium dodecyl sulfate (SDS)-containing sample buffer and stored at -20 °C. To confirm equal loading of the proteins, an initial gel was stained with Coomassie blue. SDS–polyacrylamide gel electrophoresis (PAGE) was performed on 9 or 12% polyacrylamide gels. The proteins were electrophoretically transferred onto nitrocellulose membranes (Hybond ECL RPN3032D; Amersham Pharmacia Biotech; Little Chalfont, UK) using a Bio-Rad Mini Protean II apparatus (Bio-Rad; Hercules, CA, USA). The blots were blocked with 5% milk in PBS-T (80 mM Na_2_HPO_4_, 20 mM NaH_2_PO_4_, 100 mM NaCl, and 0.1% Tween-20 at pH 7.5) for 1 h; incubated overnight at 4 °C with primary antibodies; and incubated with secondary anti-rabbit, anti-mouse, or anti-goat horseradish peroxidase-conjugated antibodies thereafter. The immunoblots were then visualized using an enhanced chemiluminescence system. Protein levels were quantified using densitometry. The relative intensities of the immunoblot signals were measured by densitometry using Scion Image for Windows software (Scion Corporation, 2000–2001. version Alpha 4.0.3.2. MD, USA) and expressed as fold-changes relative to the controls. The primary and secondary antibodies used in immunoblotting are listed in Table S1.

### Real-time polymerase chain reaction (real-time PCR)

Polymerase chain reaction analysis was performed as previously described [37]. Renal cortexes were homogenized in Trizol reagent (Invitrogen, Carlsbad, CA, USA). RNA was extracted with chloroform, precipitated with isopropanol, washed with 75% ethanol, and then dissolved in DW. The RNA concentration was determined by the absorbance at 260 nm (Ultraspec 2000; Pharmacia Biotech, Cambridge, UK). The mRNA expression of inflammatory cytokines and adhesion molecules was determined by real-time PCR. cDNA was made by reverse transcribing 5 μg of total RNA using oligo (dT) priming and superscript reverse transcriptase II (Invitrogen, Carlsbad, CA, USA). cDNA was quantified using a Smart Cycler II System (Cepheid, Sunnyvale, CA, USA) and SYBR Green was used for detection. Each PCR reaction was performed in 10 pM forward primer, 10 pM reverse primer, 2X SYBR Green Premix Ex Taq (TAKARA BIO INC, Seta 3-4-1, Japan), 0.5 μl cDNA, and H_2_O to bring the final volume to 20 μl. The relative levels of mRNA were determined by real-time PCR, using a Rotor-GeneTM 3000 Detector System (Corbette research, Mortlake, NSW, Australia). The sequences of the primers are listed in Additional file 1: Table S2.

The PCR was performed according to the following steps: 1) 95 °C for 5 min; 2) 95 °C for 20 s; 3) 58 to 60 °C for 20 s (optimized for each primer pair), and 4) 72 °C for 30 s to detect SYBR Green. Steps 2 – 4 were repeated for an additional 40 cycles, while at the end of the last cycle, the temperature was increased from 60 to 95 °C to produce a melting curve. Data from the reaction were collected and analyzed with Corbett Research Software. The comparative critical threshold values from quadruplicate measurements were used to calculate the gene expression, with normalization to GAPDH as an internal control. Melting curve analysis was performed to enhance the specificity of the amplification reaction.

### Histology

The preparation of kidney tissue proceeded as previously described [37]. Kidney tissues were fixed with 4% paraformaldehyde, embedded in paraffin, and cut into 3-μm-thick sections. Periodic acid-Schiff staining was performed according to the manufacturer’s instructions (Abcam, Cambridge, MA, USA). For immunohistochemistry, deparaffinized tissue sections were antigen-retrieved by heating at 100 ℃ for 15 min in citrate buffer, pH 6.0 (Sigma-Aldrich). The sections were incubated overnight at 4 ℃ with primary antibodies diluted in blocking buffer (1% [w/v] bovine serum albumin dissolved in 0.3% [v/v] Triton X-100 prepared in PBS [0.3% PBS-T]). After the sections were briefly washed thrice with 0.3% PBS-T, they were incubated for 1 h at RT with secondary antibodies diluted in blocking buffer. Tubular injury scores were calculated by previously described method [39, 40]. Tubular injury score was measured by examination of ≥ 10 HPF of corticomedullary junction (× 200 magnification) in PAS-stained sections (n = 5–7). The primary and secondary antibodies used in the immunohistochemical analyses are listed in Table S3. Apoptosis of tubular epithelial cells was detected with TUNEL staining with ApopTag Plus Peroxidase In Situ Apoptosis Kit (Sigma-Aldrich), according to the manufacturer’s instruction. Tissue sections of all major organs were collected paraffin embedded and were stained with hematoxylin/eosin (H&E) to compare the histological changes.

### Statistical analysis

The results are expressed as the mean ± standard error of the mean (SEM). Multiple comparisons among the three groups were performed using one-way analysis of variance (ANOVA) and the post-hoc Tukey’s honestly significant difference test. Differences with p-values of less than 0.05 were considered significant.

## Supplementary Information

**Additional file 1: Figure S1**. (A) Powder sample of prepared dMn_3_O_4_ (B) Schematic representation and (C) EDS analysis of dMn_3_O_4_. **Figure S2**. XPS analysis individual peaks in dMn_3_O_4_. **Figure S3**. NMR analysis (A) PTC and (B) PC. **Figure S4**. Low magnification FE-TEM images of (A) PTC (B) PC-M, PC-M + H_2_O_2_, PTC-M and PTC-M + H_2_O_2_. **Figure S5**. EDS elemental mapping of PTC-M. **Figure S6**. (A) Lyophilized sample of PTC-M (B) TGA analysis. **Figure S7**. (A) Hydrodynamic diameter and (B) zeta potential of PTC-M and PC-M before and after exposure to H_2_O_2_. **Figure S8**. (A) Hydrodynamic size distribution and (B) zeta potential of empty nanomicelles (PTC and PC) before and after treatment with H2O2. **Figure S9**. Stability of PTC-M in 10% FBS. **Figure S10**. Catalase like activity of PTC-M at different concentrations. **Figure S11**. (A) Disproportionation of H_2_O_2_ by PTC-M resulting in bubble formation (B) Dissolved oxygen production by dMn_3_O_4_ and PTC-M. Data is shown as mean ± SEM (n = 3 replicates). Statistics were performed by a one-way ANOVA (*P < 0.05, **P < 0.01, *** P < 0.001, and **** P < 0.0001). **Figure S12.** In vitro cell viability of the empty nanomicelles (PTC, PC) as well as dMn3O4 loaded nanomicelles (PTC-M, PC-M). **Figure S13**. Cellular uptake and intracellular IR780 release. High magnification images (Scalebar=75µm). **Figure S14**. (A) Ex-vivo images of organs at different time points post injection form I/R model mice administered with PC-IR780 and IR780 (B) NIRF signal intensity quantification from isolated kidneys of PTC-IR780, PC-IR780 and IR780 administered mice in I/R model at different time points. **Figure S15**. Ex-vivo images of major organs at different time points post injection form normal C57 mice administered with PTC-IR780. **Figure S16**. ICP-MS analysis of major organs, feces and urine collected from AKI mice administered with PTC-M at 72 h and 168h. **Figure S17**. H & E staining of all major organs (liver, lung, spleen, heart, and intestine) collected from the control and PTC-M treated AKI mice (at 72 h), scale bar = 200 μm. **Table S1**. List of primary and secondary antibodies for immunoblotting. **Table S2**. List of primer sequences for real-time qPCR. **Table S3**. List of primary and secondary antibodies for immunohistochemistry.

## Data Availability

All data generated or analyzed during this study are included in this published article and its Additional file 1. Research data are not shared.
