# Supplementary material for: Inflammation-sensing catalase-mimicking nanozymes alleviate acute kidney injury via reversing local oxidative stress
Source: J Nanobiotechnology. 2022 Apr 27;20:205. doi: 10.1186/s12951-022-01410-z (PMC9044883; doi:10.1186/s12951-022-01410-z)
Supplement: Supplementary file 1 — Additional file 1: Figure S1. (A) Powder sample of prepared dMn3O4 (B) Schematic representation and (C) EDS analysis of dMn3O4. Figure S2. XPS analysis individual peaks in dMn3O4. Figure S3. NMR analysis (A) PTC and (B) PC. Figure S4. Low magnification FE-TEM images of (A) PTC (B) PC-M, PC-M + H2O2, PTC-M and PTC-M + H2O2. Figure S5. EDS elemental mapping of PTC-M. Figure S6. (A) Lyophilized sample of PTC-M (B) TGA analysis. Figure S7. (A) Hydrodynamic diameter and (B) zeta potential of PTC-M and PC-M before and after exposure to H2O2. Figure S8. (A) Hydrodynamic size distribution and (B) zeta potential of empty nanomicelles (PTC and PC) before and after treatment with H2O2. Figure S9. Stability of PTC-M in 10% FBS. Figure S10. Catalase like activity of PTC-M at different concentrations. Figure S11. (A) Disproportionation of H2O2 by PTC-M resulting in bubble formation (B) Dissolved oxygen production by dMn3O4 and PTC-M. Data is shown as mean ± SEM (n = 3 replicates). Statistics were performed by a one-way ANOVA (*P < 0.05, **P < 0.01, *** P < 0.001, and **** P < 0.0001). Figure S12. In vitro cell viability of the empty nanomicelles (PTC, PC) as well as dMn3O4 loaded nanomicelles (PTC-M, PC-M). Figure S13. Cellular uptake and intracellular IR780 release. High magnification images (Scalebar=75µm). Figure S14. (A) Ex-vivo images of organs at different time points post injection form I/R model mice administered with PC-IR780 and IR780 (B) NIRF signal intensity quantification from isolated kidneys of PTC-IR780, PC-IR780 and IR780 administered mice in I/R model at different time points. Figure S15. Ex-vivo images of major organs at different time points post injection form normal C57 mice administered with PTC-IR780. Figure S16. ICP-MS analysis of major organs, feces and urine collected from AKI mice administered with PTC-M at 72 h and 168h. Figure S17. H & E staining of all major organs (liver, lung, spleen, heart, and intestine) collected from the control and PTC-M trea [file 12951_2022_1410_MOESM1_ESM.docx]

Supporting information for

**Inflammation-sensing catalase-mimicking nanozymes alleviate acute kidney injury by reversing local oxidative stress**

Hong Sang Choi, Ansuja Pulickal Mathew, Saji Uthaman, Arathy Vasukutty, In Jin Kim, Sang Heon Suh, Chang Seong Kim, Seong Kwon Ma, Sontyana Adonijah Graham, Soo Wan Kim_,_ In-Kyu Park* and Eun Hui Bae*

*Corresponding author: Eun Hui Bae, Email: [baedak76@gmail.com](mailto:baedak76@gmail.com); In-Kyu Park, Email: pik96@jnu.ac.kr

**This file includes:**

Figures. S1 to S17

Tables S1 to S3

**(B)**

**(A)**


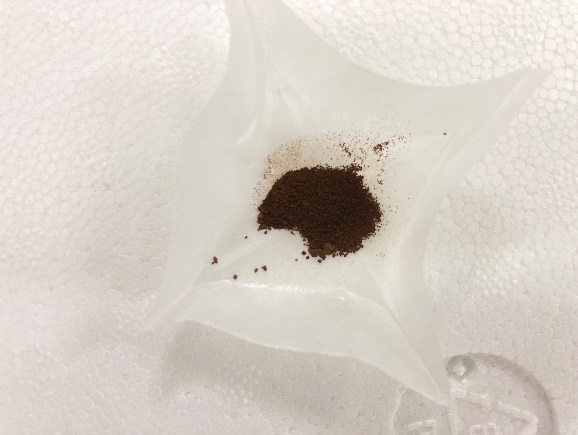

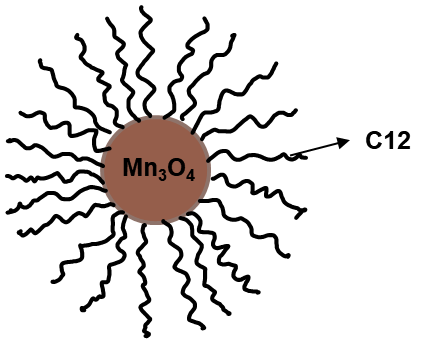

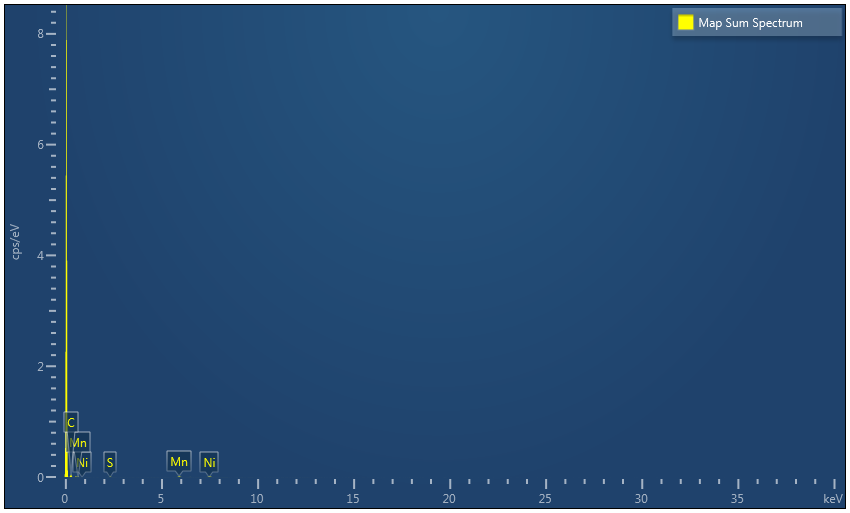


**(C)**


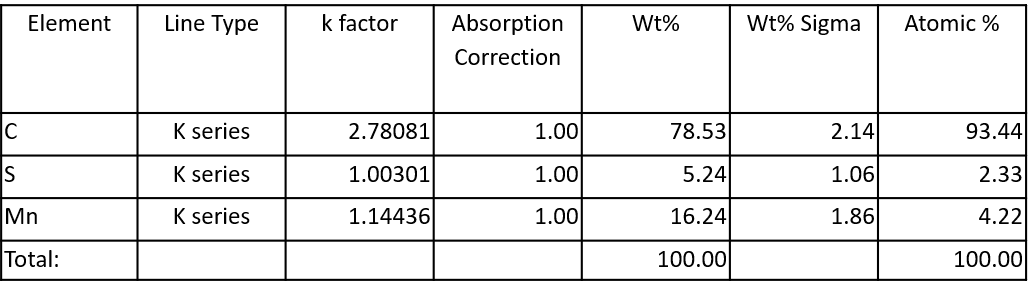


**Figure S1.** (A) Powder sample of prepared dMn_3_O_4_ (B) Schematic representation and (C) EDS analysis of dMn_3_O_4_


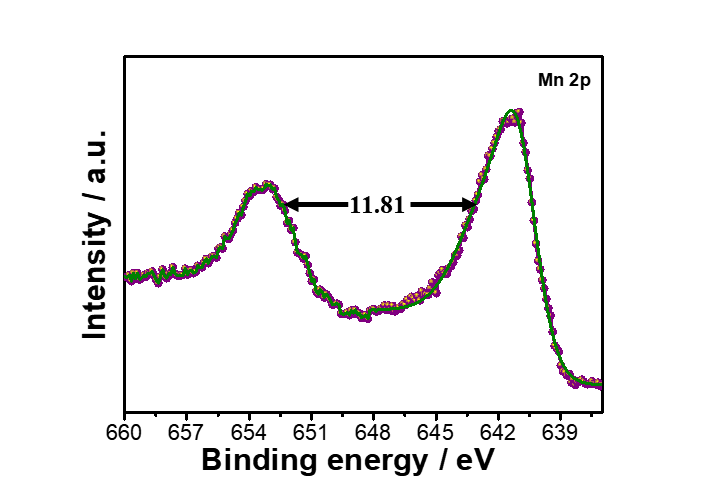


**Figure S2.** XPS analysis individual peaks in dMn_3_O_4_


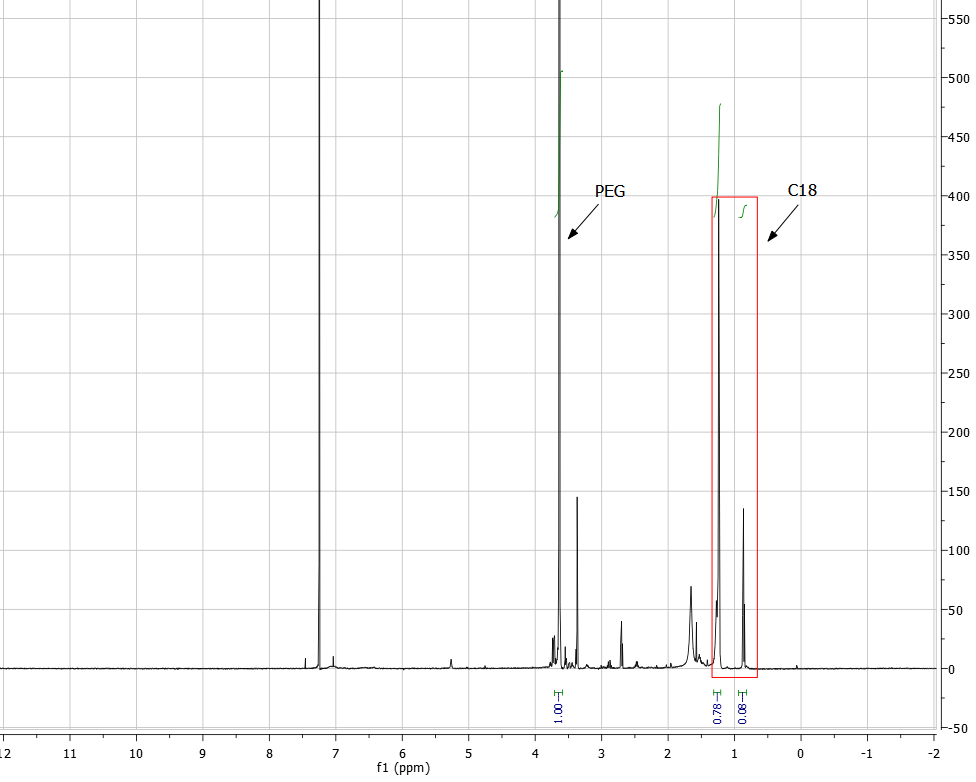

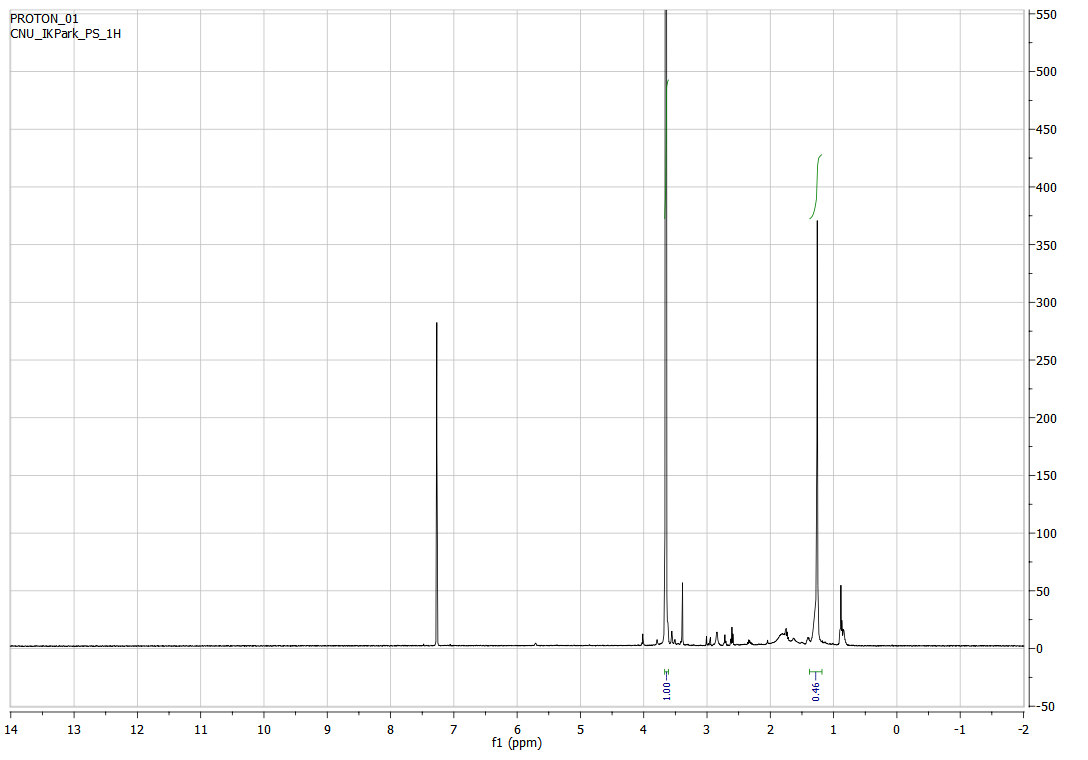


**(B)**

**(A)**

**Figure S3.** NMR analysis (A) PTC and (B) PC

**(A)**


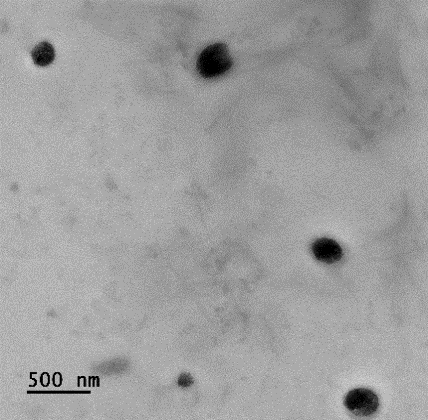


**PTC**

**(B)**

**(B)**


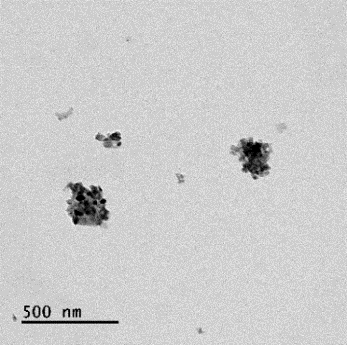

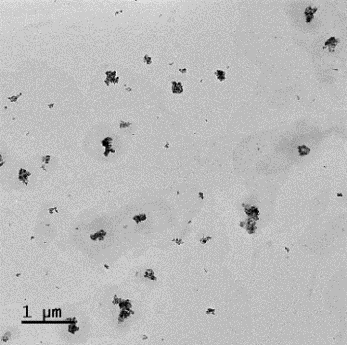

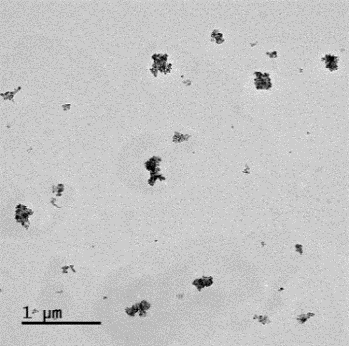

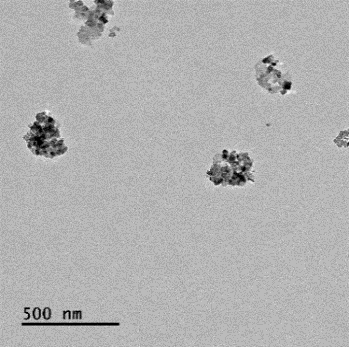

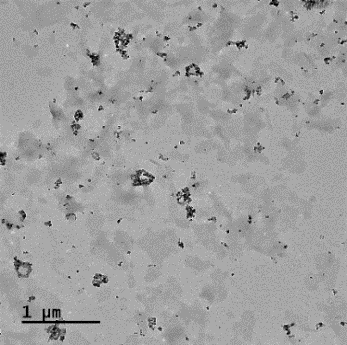

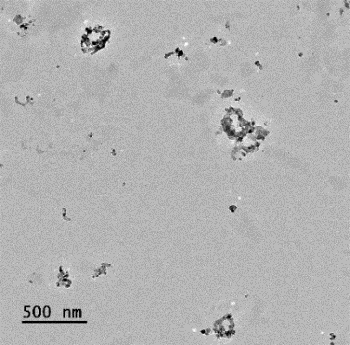


**PC-M**

**PC-M + H₂O ₂**

**PTC-M + H₂O ₂**


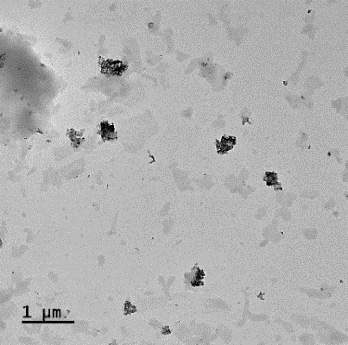

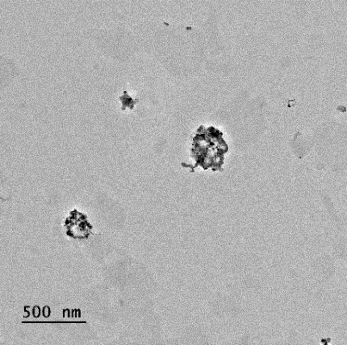


**PTC-M**

**4X**

**10X**

**Figure S4.** Low magnification FE-TEM images of (A) PTC (B) PC-M, PC-M + H_2_O_2_, PTC-M and PTC-M + H_2_O_2._


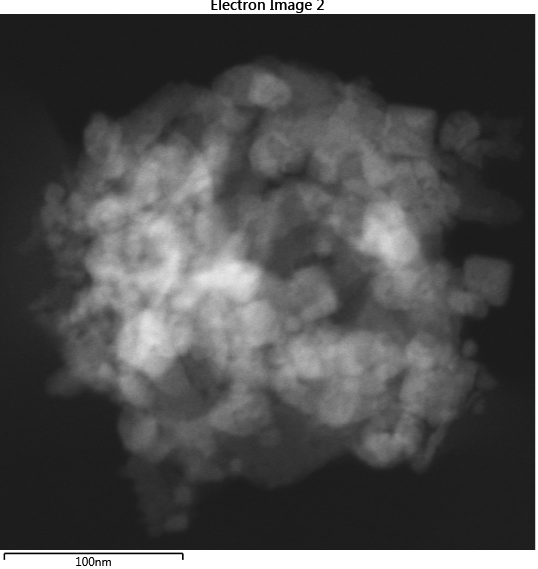

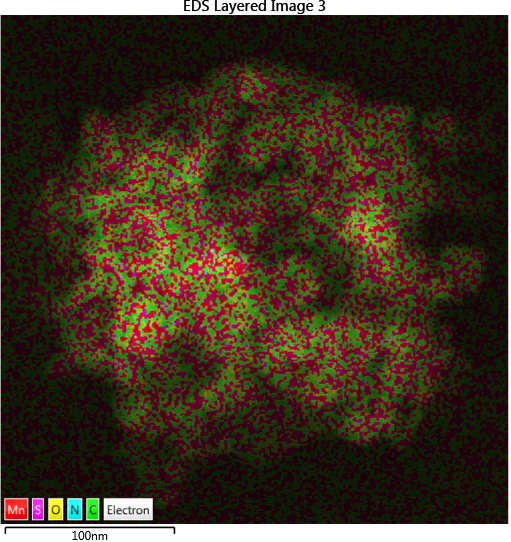

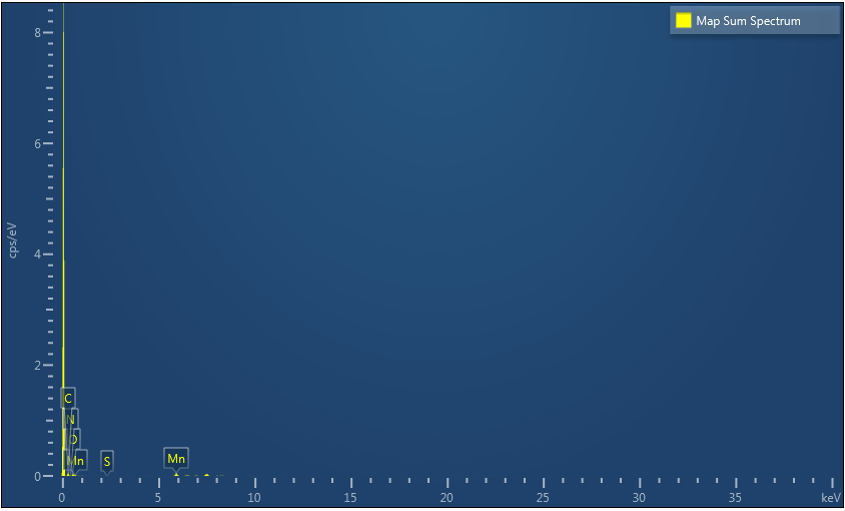

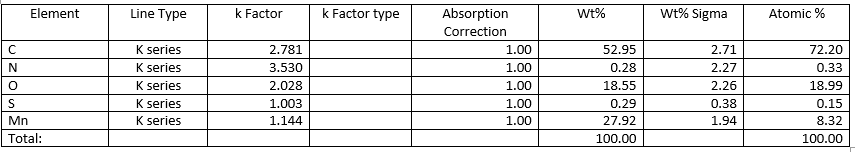


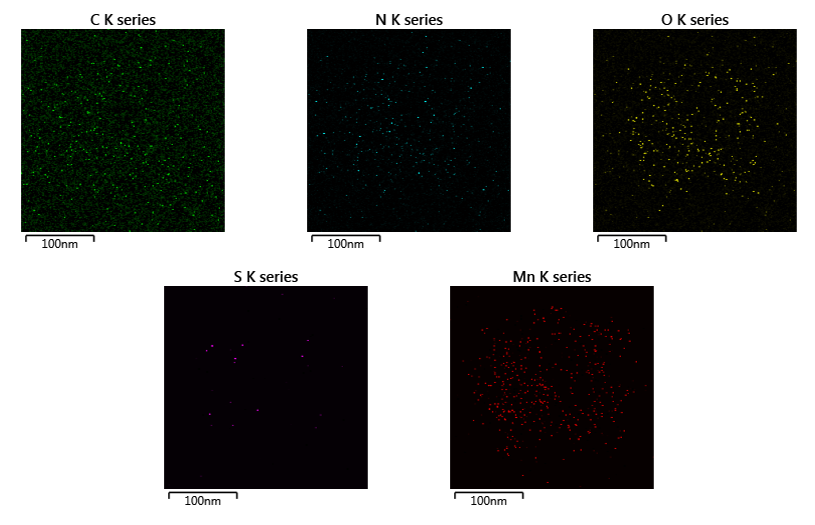

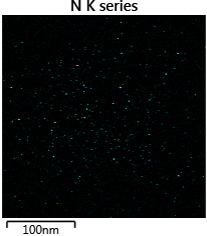

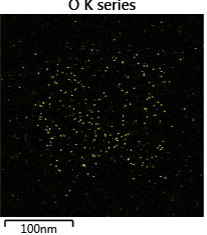

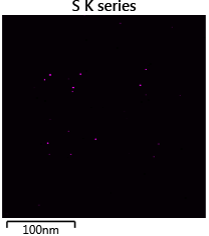

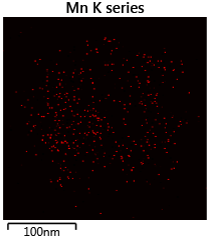


**Figure S5:** EDS elemental mapping of PTC-M


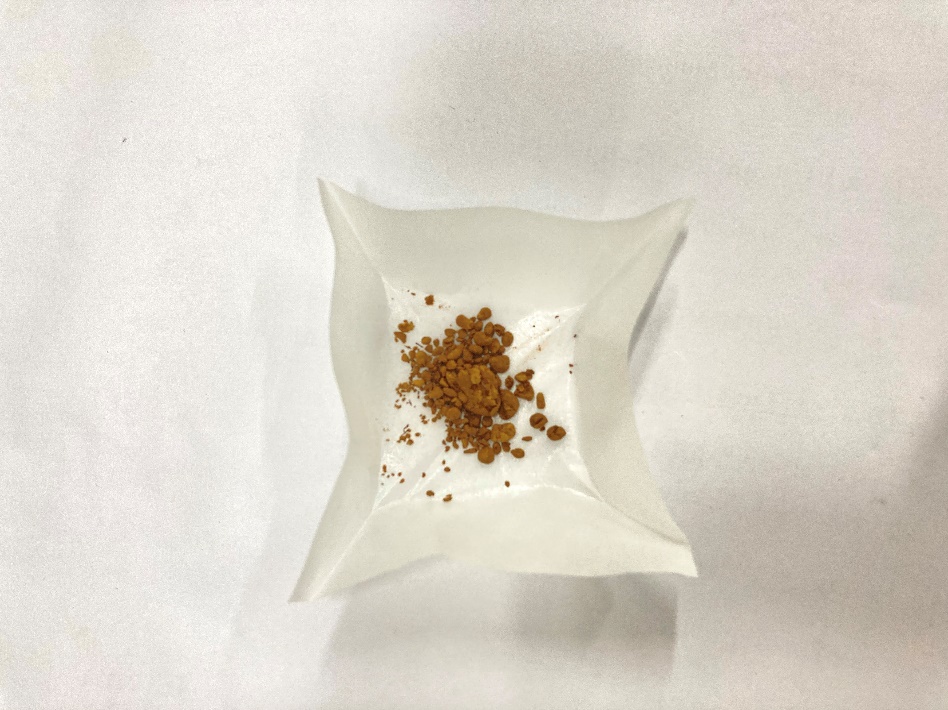


**(A)**

**(B)**


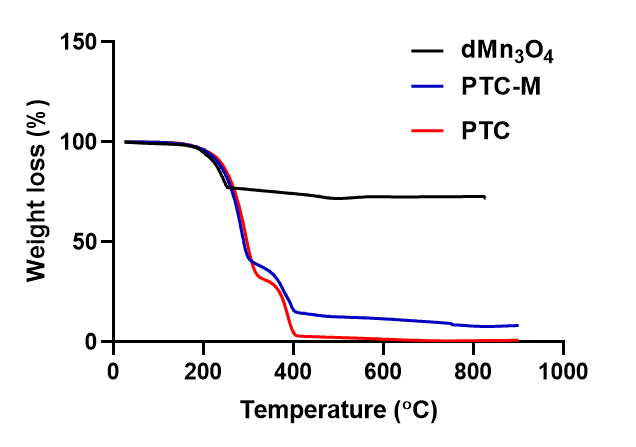


**Figure S6.** (A) Lyophilized sample of PTC-M (B) TGA analysis

**(A)**

**(B)**

**PTC-M -5.3** $\boldsymbol{\pm}\mathbf{1.8 mV}$

**PTC-M + H_2_O_2_ 4.6** $\boldsymbol{\pm}\boldsymbol{1.4}\mathbf{mV}$

**PC-M -7.2** $\boldsymbol{\pm}\mathbf{1. 8 mV}$

**PC-M + H_2_O_2_ -7.6** $\boldsymbol{\pm}\mathbf{2.5 mV}$

**Figure S7.** (A) Hydrodynamic diameter and (B) zeta potential of PTC-M and PC-M before and after exposure to H₂O₂

**(B)**

**(A)**

**PC -8.3** $\boldsymbol{\pm}\mathbf{2.1 mV}$

**PC + H_2_O_2_ -6.7** $\boldsymbol{\pm}\mathbf{1.1 mV}$

**PTC -5.0** $\boldsymbol{\pm}\mathbf{2.6 mV}$

**PTC + H_2_O_2_ 4.8** $\boldsymbol{\pm}\boldsymbol{1.5}\mathbf{mV}$

**Figure S8.** (A) Hydrodynamic size distribution and (B) zeta potential of empty nanomicelles (PTC and PC) before and after treatment with H_2_O_2_

**Figure S9.** Stability of PTC-M in 10% FBS

**Fig. S10.** Catalase like activity of PTC-M at different concentrations


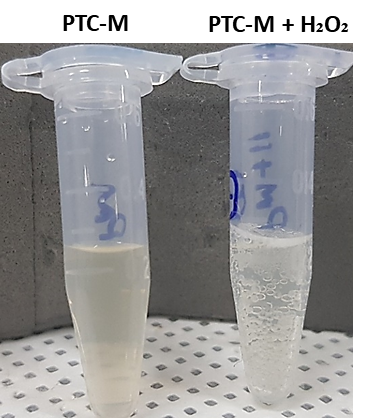


**(A)**

**(B)**

**Figure S11.** (A) Disproportionation of H_2_O_2_ by PTC-M resulting in bubble formation (B) Dissolved oxygen production by dMn_3_O_4_ and PTC-M. Data is shown as mean ± SEM (n = 3 replicates). Statistics were performed by a one-way ANOVA (*P < 0.05, **P < 0.01, *** P < 0.001, and **** P < 0.0001)

**Figure S12:** *In vitro* cell viability of the empty nanomicelles (PTC, PC) as well as dMn_3_O_4_ loaded nanomicelles (PTC-M, PC-M)


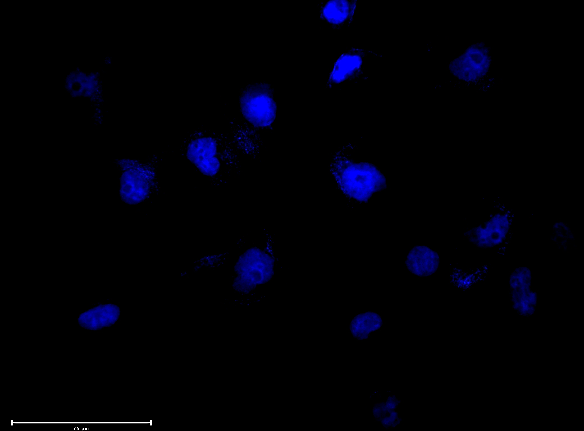

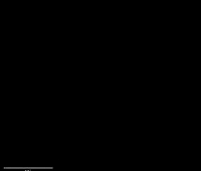

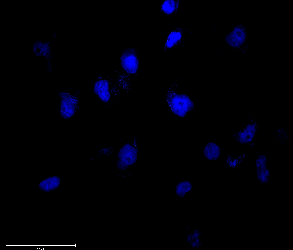


**Cell only**


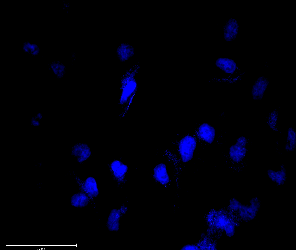

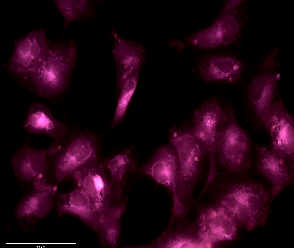

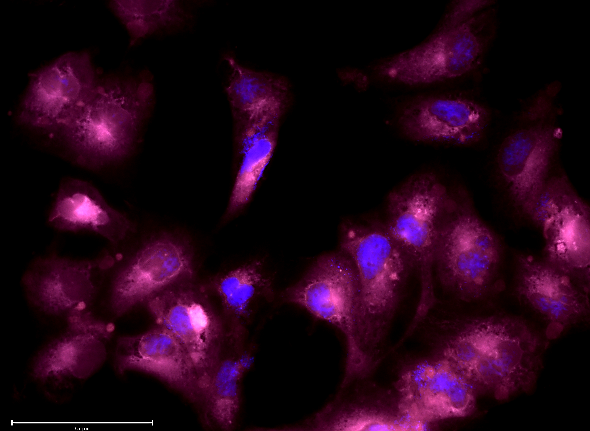


**IR 780**


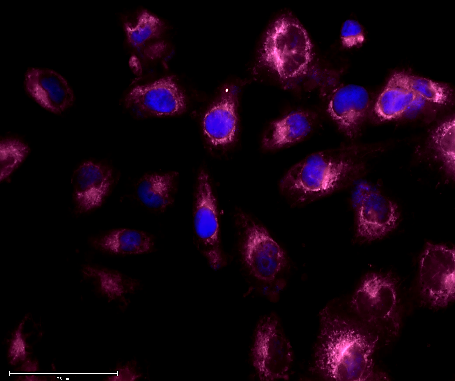

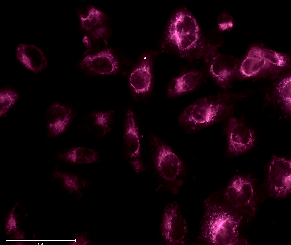

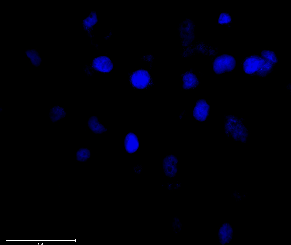

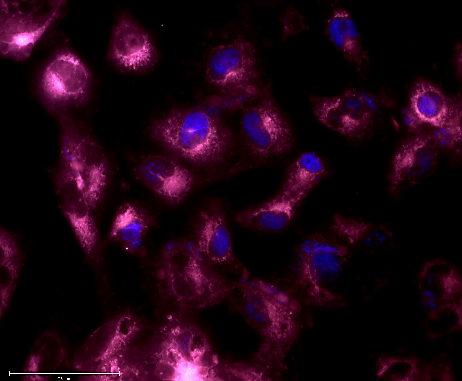

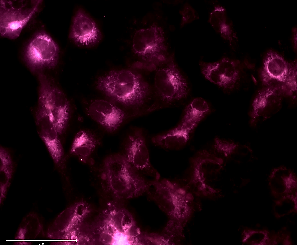

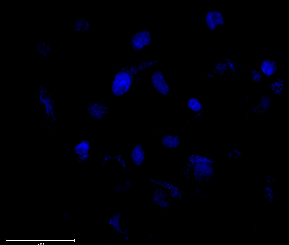


**PC-IR780**

**PTC-IR780**

**Merge**

**Merge**

**Merge**

**Merge**

**DAPI**

**Cy 7**

**Cy 7**

**Cy 7**

**Cy 7**

**DAPI**

**DAPI**

**DAPI**

**Figure S13.** Cellular uptake and intracellular IR780 release. High magnification images (Scalebar=75µm)


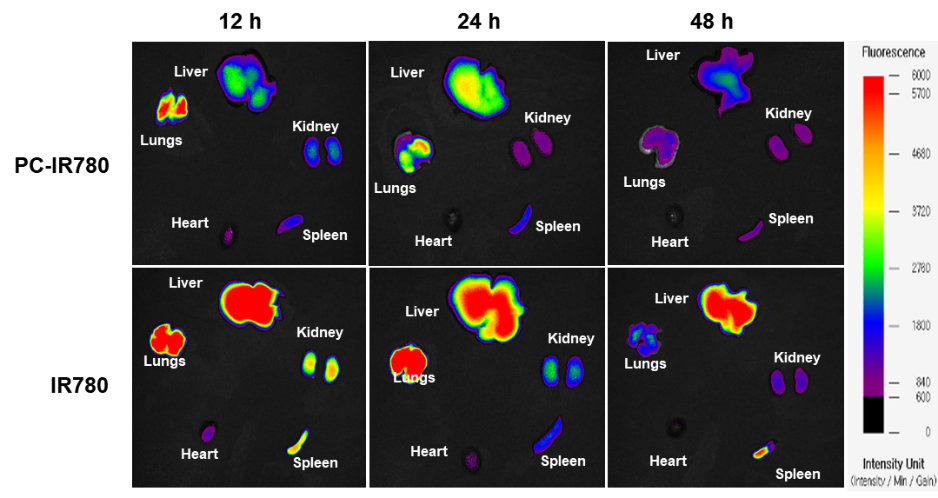


**(A)**

**(B)**

**Figure S14.** (A) *Ex vivo* images of organs at different time points post injection form I/R model mice administered with PC-IR780 and IR780 (B) NIRF signal intensity quantification from isolated kidneys of PTC-IR780, PC-IR780 and IR780 administered mice in I/R model at different time points.


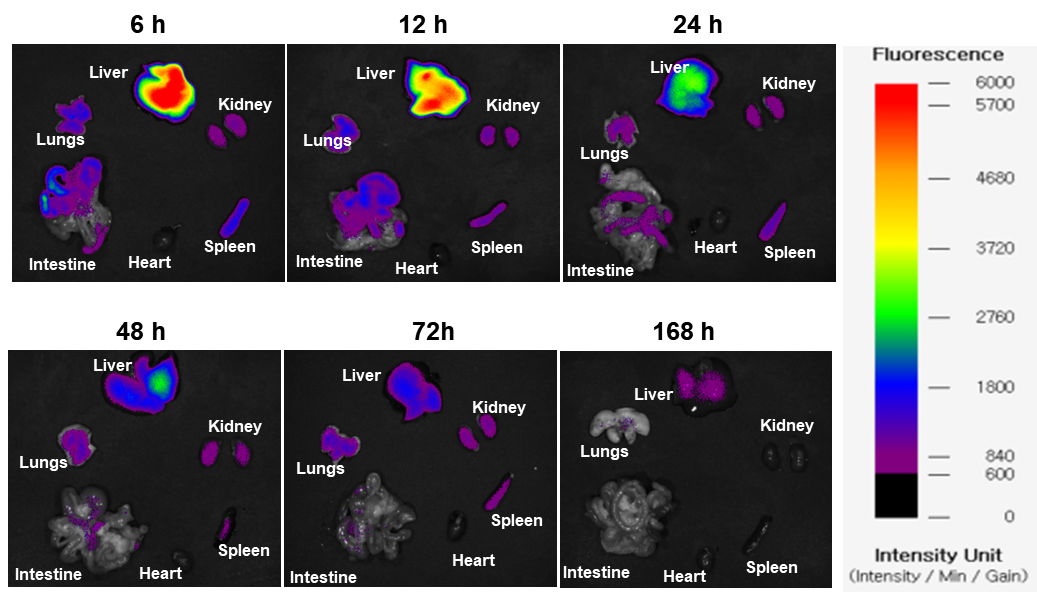


**Figure S15.** Ex-vivo images of major organs at different time points post injection form normal C57 mice administered with PTC-IR780.


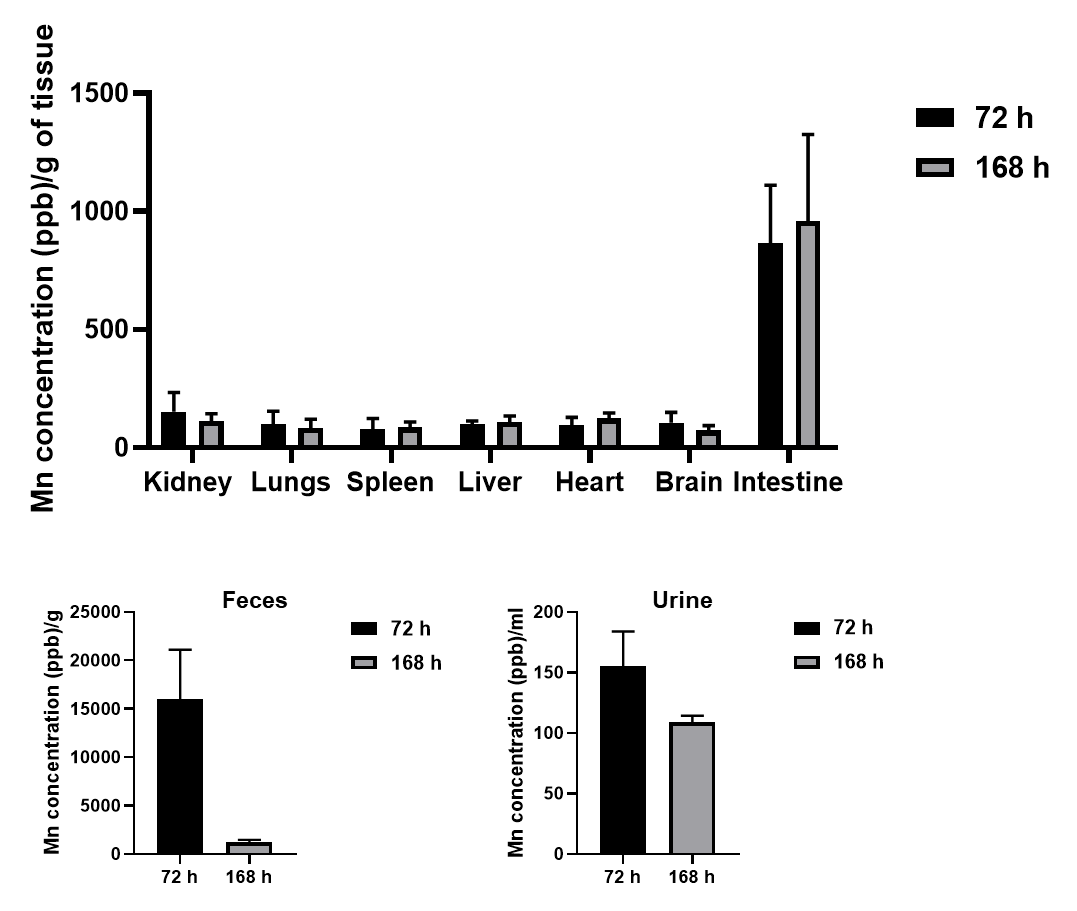


**Figure S16.** ICP-MS analysis of major organs, feces and urine collected from AKI mice administered with PTC-M at 72 h and 168h.


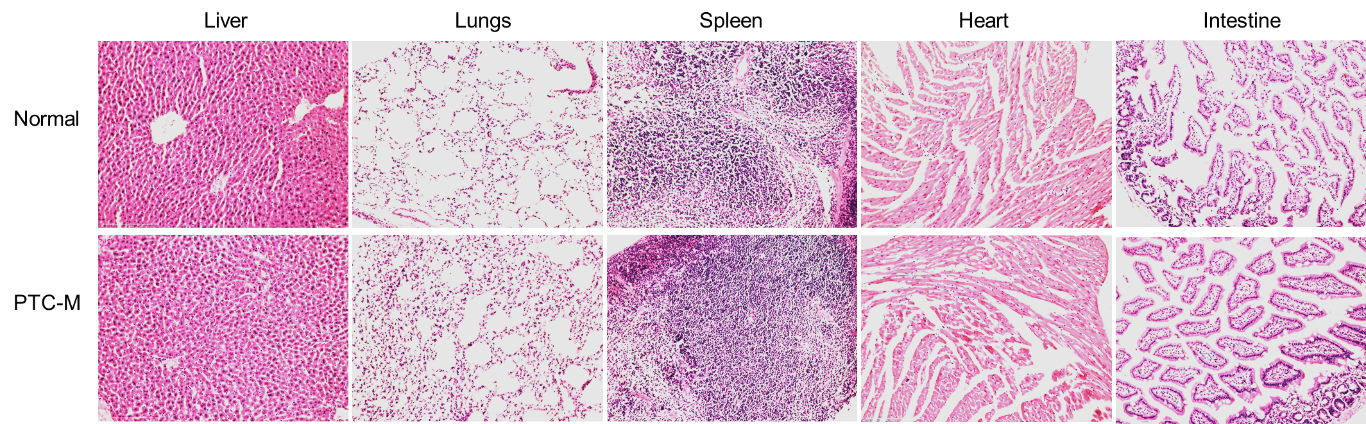


**Figure S17.** H & E staining of all major organs (liver, lung, spleen, heart, and intestine) collected from the control and PTC-M treated AKI mice (at 72 h), scale bar = 200 μm.

**Table S1**. List of primary and secondary antibodies for immunoblotting

|  | Host | Reactivity | Supplier | Cat. No. |
| --- | --- | --- | --- | --- |
| Bax | Rabbit | Human, Mouse | Cell signaling | #2772 |
| Bcl-2 | Rabbit | Human, Mouse | Cell signaling | #3498 |
| Caspase 3 | Rabbit | Human, Mouse | Cell signaling | #9662 |
| Cleaved caspase 3 | Rabbit | Human, Mouse | Cell signaling | #9661 |
| Erk1/2 | Rabbit | Human, Mouse | Cell signaling | #9102 |
| Heme oxygenase 1 | Mouse | Mouse | Abcam | ab13248 |
| JNK | Rabbit | Human, Mouse | Cell signaling | #9252 |
| p38 | Rabbit | Human, Mouse | Cell signaling | #9212 |
| Phopho-Erk1/2 | Rabbit | Human, Mouse | Cell signaling | #9101 |
| Phopho JNK | Rabbit | Human, Mouse | Cell signaling | #9251 |
| Phospho p38 | Rabbit | Human, Mouse | Cell signaling | #9215 |
| β-actin | Rabbit | Human, Mouse | Cell Signaling | #3711 |
| Goat IgG, HRP-linked | Rabbit | Goat IgG | Sigma-Aldrich | AP106P |
| Rabbit IgG, HRP-linked | Goat | Rabbit IgG | Cell Signaling | #7074 |
| Mouse IgG, HRP-linked | Horse | Mouse IgG | Cell Signaling | #7076 |

**Table S2.** List of primer sequences for real-time qPCR

|  | Forward | Reverse |
| --- | --- | --- |
| *Mus musculus* |  |  |
| *Ccl2* (MCP-1) | ATCCCAATGAGTAGGCTGGAGAGC | CAGAAGTGCTTGAGGTGGTTGTG |
| *Gapdh* | TGTGTCCGTCGTGGATCTGA | GATGCCTGCTTCACCACCTT |
| *Icam1* | AACTTTTCAGCTCCGGTCCTG | TCAGTGTGAATTGGACCTGCG |
| *Il-6* | ACAACCACGGCCTTCCCTACTT | CACGATTTCCCAGAGAACATGTG |
| *Ccl2* (MCP-1) | ATCCCAATGAGTAGGCTGGAGAGC | CAGAAGTGCTTGAGGTGGTTGTG |
| *Tgfb1* (TGFβ) | CAACAATTCCTGGCGTTACCTTGG | GAAAGCCCTGTATTCCGTCTCCTT |
| *Tnf* (TNFα) | GCATGATCCGCGACGTGGAA | AGATCCATGCCGTTGGCCAG |
| *Vcam1* | TCTCTCAGGAAATGCCACCC | CACAGCCAATAGCAGCACAC |

**Table S3.** List of primary and secondary antibodies for immunohistochemistry

|  | Host | Reactivity | Supplier | Cat. No. |
| --- | --- | --- | --- | --- |
| F4/80 | Rat | Mouse | Bio-rad | MCA497GA |
| HO-1 | Mouse | Mouse | Abcam | ab13248 |
| Rabbit IgG, HRP-linked | Goat | Rabbit IgG | Vector | PI-1000 |
| Rat IgG, HRP-linked | Goat | Rat IgG | Vector | PI-9400 |
| Mouse IgG, HRP-linked | Goat | Mouse IgG | Vector | PI-2000 |
